# Measuring Resilience in the Context of Conflict-Related Sexual
Violence: A Novel Application of the Adult Resilience Measure
(ARM)

**DOI:** 10.1177/08862605211028323

**Published:** 2021-07-07

**Authors:** Janine Natalya Clark, Philip Jefferies, Sarah Foley, Michael Ungar

**Affiliations:** 1 University of Birmingham, UK; 2 Dalhousie University, Halifax, Nova Scotia, Canada; 3 University of Edinburgh, UK

**Keywords:** conflict-related sexual violence, resilience, Adult Resilience Measure (ARM), resources, protective factors, cultural contexts, social ecologies

## Abstract

There is a rich body of research addressing the issues of conflict-related sexual
violence, and a similar wealth of scholarship focused on resilience. To date,
however, these literatures have rarely engaged with each other. This article
developed from an ongoing research project that seeks to address this gap, by
exploring how victims-/survivors of conflict-related sexual violence in three
highly diverse settings – Bosnia-Herzegovina, Colombia and Uganda – demonstrate
resilience. This research is the first to apply the Adult Resilience Measure
(ARM), a 28-item scale that seeks to measure protective resources across
individual, relational, and contextual subscales, to the context of
conflict-related sexual violence. A total of 449 female and male participants in
the three aforementioned countries completed the ARM (in the framework of the
study questionnaire) as part of this research. This article presents some of the
results of the analyses. Specifically, we first sought to establish through
Confirmatory Factor Analysis whether the ARM was actually measuring the same
construct in all three countries, by confirming the invariance (or otherwise) of
the factor structure. The second aim was to explore how different resources
function and cluster in different cultural contexts, to arrive at a more nuanced
understanding of the different protective factors in the lives of study
participants. We generated different factor structures for BiH, Colombia, and
Uganda respectively, suggesting that a single factor structure does not
sufficiently capture the diverse groupings of protective factors linked to the
particularities of each country, including the dynamics of the conflicts
themselves. Ultimately, we use the findings to underscore the need for policy
approaches that move away from a deficit model and give greater attention to
strengthening and investing in the (often overlooked) protective resources that
victims-/survivors may already have in their everyday lives.

## Introduction

Spanning multiple disciplines, there exists a wealth of literature addressing
resilience (see, e.g., Folke, 2016; Ungar, 2019; Chandler, 2020; Quinn et al., 2020). Within scholarship on conflict-related sexual violence, however,
resilience remains a significantly overlooked and underexplored concept. Part of the
explanation arguably lies in the prevalence of what Tuck (2009, p. 413) has referred to as
“damage-centered research.” Although she uses the term in relation to research with
Indigenous communities, it also resonates in the context of work on conflict-related
sexual violence. A strong emphasis on “damage” and harm done to those subjected to
such violence (see, e.g., Durbach & Chappell, 2014; Ba & Bhopal, 2017) frequently detracts
from other dimensions of their experiences. In this regard, the lack of attention to
resilience is one example of the “incomplete story” that Tuck (2009, p. 416) associates with
damage-centered research.

This article developed out of an ongoing five-year research project that seeks
precisely to offer a more “complete story” about conflict-related sexual violence
through its focus on resilience. Specifically, it is examining how
victims-/survivors^1^ of such violence (and other interrelated/co-occurring forms
of violence) demonstrate resilience in their daily lives, how their particular
environments shape and enable expressions of resilience, and how cross-contextual
factors that support resilience operate in different settings. It is accordingly
using three maximum diversity case studies—namely Bosnia-Herzegovina (BiH),
Colombia, and Uganda—that reflect significant variation across key social–ecological
variables, including family structure, institutional resources, and cultural
systems. All three countries have experienced high levels of conflict-related sexual
violence (including rape, forced nudity, sexual torture, and genital mutilation) in
the context of different conflict dynamics over different time scales.

Consistent with a broad shift in resilience scholarship away from person-centric,
psychological explanations toward a focus on inter-connected social–ecological
systems (see, e.g., Berkes et al., 2003; Folke, 2006), this article and the underpinning research study locate resilience
in the interactions between individuals and their wider social ecologies. Discussing
resilience and children, for example, Ungar (2011, p. 6) argues that “the
child’s own individual resources (e.g., a sense of humor, optimism, above average
IQ, or musical talents) are only as good as the capacity of his or her social and
physical ecologies that facilitate their expression and application to developmental
tasks.” These ecologies—including family, community, and institutions—and the
support and resources that they offer are similarly crucial when thinking about
resilience in the context of conflict-related sexual violence (Clark, 2021a). This article thus
understands resilience as “the qualities of both the individual and the individual’s
environment that potentiate positive development” (Ungar & Leibenberg, 2011, p. 127).

This definition, in turn, makes it clear that resilience is a highly “dynamic, fluid
process” (Henshall et al., 2020, p. 3598), which necessarily raises important measurement issues.
Indeed, “there is no universally accepted methodology for operationalizing and
measuring resilience empirically” (Alessi et al., 2020, p. 570). For this
purpose, we ultimately chose to use the Adult Resilience Measure, or ARM (Resilience Research Centre, 2016), a 28-item scale divided into individual, relational, and
contextual sub-scales. Our reasons for using the ARM over other resilience scales,
including the Connor-Davidson Resilience Scale (Connor & Davidson, 2003) and the Brief
Resilience Scale (Smith et al., 2008), were twofold. First, the ARM reflects a social–ecological approach
to resilience, focusing not just on individual assets but also on the capacity of
people’s environments to provide the resources that individuals require to cope when
exposed to atypical amounts of stress or adversity. Each item in the scale is scored
on a 5-point scale and overall ARM scores (ranging from 28 to 140) are an indicator
of the protective resources that people have in their lives to support
resilience.

Second, we wanted to use a measurement tool that we felt could be easily understood
in BiH, Colombia, and Uganda, including by participants with little or no education.
A notable strength of the ARM is that it is an adaptation of the Child and Youth
Resilience Measure (CYRM), the development of which involved multiple cross-cultural
research sites (Ungar & Liebenberg, 2011, p. 134). Also highly relevant is Liebenberg and Moore’s (2018, p. 13)
finding, based on their own use of the ARM, that “in contrast to some longer and
more complex measures of resilience, the RRC [Resilience Research Centre]-ARM may be
a good fit for vulnerable adult populations.”

Liebenberg and Moore (2008) utilized the ARM in their research on Irish survivors of clerical
institutional abuse. The measure has also been used, *inter alia*, in
a study exploring resilience as a moderator of substance use outcomes in the context
of young adults (Kurtz et al., 2019) and in a study about post-traumatic stress disorder (PTSD) in war
veterans and civilians (Wall & Lowe, 2020). This research is the first to use the ARM in relation
to victims-/survivors of conflict-related sexual violence—and the first to apply it
to a comparative study of BiH, Colombia, and Uganda. Significant in this regard is
Daigneault et al.’s (2013, p. 161) observation that “The search for a singular metric with an
invariant factorial structure across the globe may be fruitless, as the meaning of
resilience likely varies according to context… .” The first aim of this research,
thus, was to establish through confirmatory factor analysis (CFA) whether the ARM
was actually measuring the same construct in all three countries, by confirming the
invariance (or otherwise) of the factor structure. The second aim was to explore how
different resources function and cluster in different cultural environments, to
arrive at a more nuanced understanding of the different protective factors in the
lives of study participants.

## Resilience in the Context of Conflict-related Sexual Violence

Gopal and Nunlall (2017,
p. 63) note that “Although much research has been reported on the nature and trends
of violence against women, few studies have focussed on what may be regarded as
necessary for their ‘survival’ during and post-violence.” Particularly in the
context of extant scholarship on conflict-related sexual violence, what also stands
out is a lack of attention to the various ways that those who have experienced such
violence “survive” in the sense of rebuilding and moving forward with their lives—in
interaction with their social ecologies. When scholars writing about
conflict-related sexual violence have referred to resilience (see, e.g., Zraly et al., 2013; Koos, 2018), they have
tended to do so in a very abstract and peripheral way that does not substantively
engage with the concept, its meaning or its complexity.^2^ As one illustration, a
Ugandan-based study by Edström et al. (2016, p. 5) finds that “despite pervasive discrimination, groups
of male survivors have been able to develop resilience and mutual support through
collective action.” At no point, however, do the authors actually define
resilience.

Related concepts are also underexplored. Hope, for example, can contribute to
resilience, in the sense of giving people a reason to get on with their lives and
engage in processes that support a future orientation (Eggerman & Panter-Brick, 2010, p. 72).
Yet hope has received little attention in discussions about conflict-related sexual
violence, and so too have the goals and desires that help motivate individuals to go
forward (Wallström, 2012, p. 5). Indeed, citing a victim-/survivor in the Democratic Republic of
Congo, Dossa et al. (2014, p. 249) suggest that “a raped woman is no longer capable of
pursuing her dreams because of how she is regarded in her community.”

It is impossible to write about resilience without acknowledging and discussing some
of the trenchant criticisms that it has attracted. Indeed, the many arguments
problematizing the concept may further help to explain why resilience has not
received the attention that it arguably merits in research on conflict-related
sexual violence. Critical voices have particularly focused on wider normative and
ideological issues. Joseph (2013, p. 40), for example, maintains that resilience “has been plucked
from the ecology literature and used in a fairly instrumental way to justify
particular forms of governance which emphasize responsible conduct,” meaning that
individuals are expected “to govern themselves in appropriate ways” (see also Chandler, 2014; Welsh, 2014). A related
concern, illustrating Chandler’s (2020, p. 210) discussion of “artificial” or “coercive” forms
of adaptation, is that some individuals and communities have no choice but to be
resilient in the face of shocks and stressors that are both unequally distributed
and reflective of deeper inequalities and power imbalances. For example, Smyth and Sweetman (2015,
p. 410) point out that “Women living in poverty in contexts threatened by complex
crises are required each day to be resilient and withstand stresses and shocks which
threaten the wellbeing – and sometimes the very lives – of themselves and their
dependents.” The issue of “differential access to resources” (Jordan, 2019, p. 168) also has important
implications for resilience and further highlights underlying structural issues
which, according to some critics, have not been sufficiently addressed or
acknowledged within resilience scholarship (see, e.g., MacKinnon & Derickson, 2012, p. 254;
Mayor, 2018, p.
209).

In view of such critiques, which must be taken seriously, it is imperative to stress
from the outset that the underpinning research on which this article draws is not
seeking to argue that victims-/survivors of conflict-related sexual violence
*should* demonstrate resilience. Nor is its intention to deflect
attention from, or to diminish, the responsibilities that governments have toward
populations affected by conflict and violence. In addressing the neglect of
resilience within extant scholarship on conflict-related sexual violence, what it is
seeking to show is that the concept has an important and legitimate place within
this corpus of literature. Framing resilience as a social–ecological concept and
focusing on three very different societies that have experienced large-scale
violence and instability, this research is essentially seeking to gain deeper
insights into how and where environments are “succeeding” and “failing.” More
specifically, it aims to show that because “resilience does not occur in isolation”
and is “dependent upon context or environment, including our relationships” (Kent, 2012, p. 111),
exploring the factors and resources that support and enable resilience potentially
provides a basis for more contextually-sensitive interventions, including
transitional justice interventions (Clark, 2021b).

## Study Design and Methodology

### The Participants

The study sample consisted of 449 participants (*n* = 126 in BiH,
*n* = 171 in Colombia, and *n* = 152 in
Uganda), all of them victims-/survivors of conflict-related sexual violence, who
completed a questionnaire between May and December 2018. There were no exclusion
criteria, but all participants had to be aged 18 years or over and able to give
informed consent.^3^ The challenging nature of the research and the fact that
there are no publicly available lists of victims-/survivors of conflict-related
sexual violence in any of the three countries, for obvious reasons, meant that
it was largely necessary to rely on a convenience sampling strategy. This
involved close collaboration with several in-country organizations^4^ that are
supporting the research and facilitated crucial access to research participants.
Some of these organizations were working directly with victims-/survivors of
conflict-related sexual violence. Others had links to them through their
existing contacts. The organizations also played a role in verifying that the
participants had suffered conflict-related sexual violence. Further evidence of
this was the fact that some of the participants, particularly those in BiH, had
testified in court against their accused. In addition, some of the Bosnians were
in receipt of monthly payments from the state (as a form of compensation [see
Clark, 2017,
chapter 6]); and some of the participants in Colombia had received reparations
from the country’s Victims’ Unit.

Individuals that have contact with local organizations might be assumed to have
greater access to resilience-supporting resources than those who do not have
such contact, thus potentially creating a bias within the sample. However, two
important points should be underlined in this regard. First, some of the
participants were merely known to the individual in-country organizations and
were not necessarily in regular contact with them or in receipt of any direct
support. Indeed, many of them had not received any help, particularly some of
the participants living in remote areas of northern Uganda, and this was one of
the reasons why a referral network was built into the study design (discussed
below under “Ethics Issues”). Second, even when participants did have close
contact with one of the organizations, it is important not to automatically
assume that these relationships exerted more influence on participants’
resilience than their relationships with other parts of their social ecologies,
including their families, children, friends, and faith.

Convenience sampling was combined with elements of purposive sampling, meaning
that particular categories of victims-/survivors were specifically sought out to
ensure that the samples, as much as possible, captured some of the demographic
variation within each country. This was also a key part of the study’s
commitment to diversity. One of its aims in this regard was to ensure that male
victims-/survivors were represented. This was important because although a
growing body of literature in recent years “has begun to recognize that sexual
violence against men and boys is perpetrated more frequently than has been
commonly assumed,”^5^ the issue nevertheless “remains underexplored in scholarship
and policymaking alike” (Schulz & Touquet, 2020, p. 1175). Finding such men, however, is
often extremely difficult, not least because some of them may have never
acknowledged what happened to them. According to Schulz (2018, p. 588), “male survivors
themselves frequently choose to remain silent, due to shame and fear of
stigmatization, as preserving the silence can be protective.” Reflecting these
challenges, only 27 (6%) of the study participants were men (12 in BiH, five in
Colombia, and 10 in Uganda).

Another diversity-led priority was to address the fact that within each country,
particular ethnic groups have received little attention, namely Serbs and Croats
in BiH, Indigenous people in Colombia and Lango people in Uganda. The challenges
of reaching some of these groups were substantial. In BiH, for example,
nongovernmental organizations (NGOs) that work with victims-/survivors of
conflict-related sexual violence committed during the Bosnian war overwhelmingly
work with Bosniak women. This makes it extremely difficult to gain access to
individuals from other ethnic groups, particularly given the general lack of
cooperation between NGOs in the BiH Federation and Republika Srpska (the
country’s two entities). Even though some ethnic groups are underrepresented in
the sample, the overall result is a unique dataset that captures some of the
complex ethnic dimensions of the conflicts in each country (see Table B.1).

Achieving diversity meant applying the study questionnaire in multiple locations
in all three countries. In BiH, questionnaires were completed in seven of the
ten cantons within the BiH Federation, in twelve different locations in
Republika Srpska, and in two locations in Brčko District (a self-governing
administrative unit). In Colombia, the application of the questionnaire covered
18 different departments, including Bolívar in the Caribbean region, Putumayo in
the Amazon region, and Antioquia in the Andean region. In northern Uganda,
research participants were located in 12 different districts in the Acholi and
Lango sub-regions, including Gulu, Pader, and Oyam. Decisions about where to
apply the study questionnaire were influenced by four key considerations:
security issues (particularly in Colombia); achieving a balance between urban
and rural locations; the clustering of conflict-related sexual violence cases in
particular areas; and on-the-ground resources (in the sense of the logistical
support that the in-country organizations could offer).

The age of participants ranged from 18 to 80 years. On average, the participants
from BiH were older overall (*M* = 55), compared to those in
Colombia (*M* = 42) and Uganda (*M* = 40). Part of
the explanation for these age variations lies in the conflicts themselves. The
majority of the Bosnian participants suffered sexual violence in 1992, the first
year of the Bosnian war. In contrast, the very protracted nature of the armed
conflict in Colombia, extending over more than 50 years, meant that Colombian
participants’ experiences of sexual violence had a much greater temporal spread.
In Uganda, some of the participants were very young when they suffered
conflict-related sexual violence; they were abducted as children and forcibly
recruited into Joseph Kony’s rebel Lord’s Resistance Army.

### Ethics Issues

The study necessarily raises many complex ethics issues, and the process of
securing ethics approval from the host institution, the research funder, and
relevant authorities in BiH, Colombia, and Uganda took many months. Issues that
needed to be comprehensively addressed included informed consent,
confidentiality, incidental findings, potential re-traumatization of research
participants, data storage, data transfer, and fair benefit sharing. It is
beyond the scope of this article to cover all of these. However, it is important
to stress that the guidelines of the World Health Organization (WHO) on
researching violence against women—which underline the four key principles of
respect for persons, maleficence (minimizing harm), beneficence (maximizing
benefits), and justice (Ellsberg & Heise, 2005, p. 36)—were closely followed. The
guidelines state, *inter alia*, that “In the case of adult women,
there is consensus among most researchers that the principles of autonomy and
confidentiality should prevail and that researchers should do everything within
their power to avoid usurping a woman’s right to make autonomous decisions about
her life” (Ellsberg & Heise, 2005, p. 37). Participants were made aware during the informed
consent process that all of the data they provided would be treated as strictly
confidential and that confidentiality would only be breached in exceptional
circumstances, namely if the researchers felt that an individual was at risk of
serious harm. Confidentiality was only breached on one occasion (and with the
participant’s consent); this particular participant had talked about wanting to
harm herself. No names were used in the research and the questionnaire did not
contain any identifying information; only the participants’ unique ID numbers
(consisting of the initials of the country, the initials of the
person/organization that administered the questionnaire and the number of the
questionnaire) were recorded. The researchers used laptops with full disk
encryption and all of the research materials were uploaded as encrypted files
(including by the in-country organizations) onto the University of Birmingham’s
extremely secure BEAR DataShare system.

The aforementioned WHO guidelines also state that “At a minimum…researchers have
an ethical obligation to provide a respondent with information or services that
can help her situation. In areas where specific violence-related services are
available, research teams have developed detailed directories that interviewers
can use to make referrals” (Ellsberg & Heise, 2005, p. 40). All participants were provided
with a participant information booklet with names and contact details of
relevant local organizations and potential sources of support. All participants,
after completing a questionnaire, received a follow-up telephone call a few days
later, in many cases from a psychologist from the nearest in-country
organization. Those who needed it were offered support by the organization or,
in some cases, were referred to external sources of support.

Participants were not paid for their involvement in the study; this might have
unduly influenced their decision to take part in it, thus compromising the
informed consent process. However, travel expenses were reimbursed and those who
had traveled longer distances (this was sometimes necessary in Colombia for
security reasons) were given lunch/refreshments.

## Measures

In addition to sociodemographic questions including age, ethnicity, marital status,
number of children, education, place of residence (e.g., city, town, village), and
employment status, the study questionnaire consisted of several measures broadly
addressing risk variables and indicators of well-being, the piloting and validation
of which are discussed below. Of particular significance was the aforementioned ARM
(*α* = .77-.95, May-Chahal et al., 2012; Liebenberg & Moore, 2018), which asks respondents to rate the extent to which they agree with
28 items using a 5-point scale (1 = “*Not at all*,” 2 = “*A
little*,” 3 = *“Somewhat*,” 4 = *“Quite a
bit*,” 5 = “*A lot*”). These items include “My family
have usually supported me through life” and “I know where to get help in my
community” (for all of the scales used, see Appendix C).

We also used a *Traumatic Events Checklist* (TEC). Different versions
of such checklists exist, including the Traumatic Experiences Checklist (Nijenhuis et al., 2002),
the War Experiences Checklist (Amone-P’Olak et al., 2007), and the Gaza Traumatic Events Checklist
(Thabet et al., 2009). To gain an overview of participants’ distressing experiences (which
extended beyond conflict-related sexual violence), the research team developed their
own TEC based on knowledge of the three conflicts and the first author’s work in BiH
spanning more than 10 years. Specifically, 20 potentially traumatic
situations—relevant to all three countries—were read out to participants and they
were asked to indicate “No,” “Yes” or “Prefer not to say” to each one. The
situations included “Been forcibly displaced from your home/community,” “Been
seriously injured/wounded” and “Had members of your family killed” (score range
0-20). The items in the TEC specifically related to war/armed conflict. However,
they necessarily covered different temporal periods, reflecting the aforementioned
fact that the duration of the conflicts in BiH, Colombia and Uganda varied
significantly (three years in BiH [1992-1995); more than five decades in Colombia
[starting in 1964]; and two decades in Uganda [1986-2006]).

Participants also completed the seven-item short-form *Centrality of Event
Scale* (CES; Berntsen & Rubin, 2006, p. 220), which measures “the extent to which
a memory for a stressful event forms a reference point for personal identity and for
the attribution of meaning to other experiences in a person’s life.” Substantial
positive correlations between high centralizing and PTSD symptoms have been found
(Berntsen & Rubin, 2006, p. 220). Although, to reiterate, participants had experienced
multiple distressing events, we used the CES to capture the centrality of the
experience/s of conflict-related sexual violence in participants’ lives (e.g., “I
feel that this event has become part of my identity” and “I feel that this event has
become a central part of my life story”). Scores ranged from 7 to 35.

Participants also completed a 12-item *Consequences of Sexual
Violence* scale, the development of which was informed by the first
author’s previous research (2017) on the long-term consequences of conflict-related
sexual violence. Using yes/no responses, participants were asked about the impact of
the sexual violence that they had experienced, including difficulties trusting other
people, broken relationships, and low self-esteem. Scores ranged from 0 to 12 and
higher scores indicated a greater number of consequences.

In addition, participants responded yes/no to 18 items reflecting current life
problems, for example experiencing economic insecurity, loneliness, and domestic
violence (drawn from researcher knowledge of the different country contexts). Four
further questions, using a 5-point scale, enquired about an individual’s quality of
life (QoL), their perceived health, how safe they felt in their community, and how
able they felt to ask for help. Higher scores indicated more positive perceptions.
Concepts such as QoL are necessarily complex, particularly in a cross-cultural
context, and several validated scales exist, including the Quality of Life Scale
(Flanagan, 1982) and
the Quality of Life Inventory (Frisch et al., 1992). Indeed, Gill and Feinstein (1994, p. 624) maintain
that “Since the 1970s, the measurement of quality of life has grown from a small
cottage industry to a large academic enterprise.” One of the priorities, however,
was to ensure that the questionnaire did not become overly lengthy and take
participants away from their everyday activities for longer than necessary. We
therefore used a single item for measuring QoL (as well as health), following the
example of some other studies (see, e.g., Siebens et al., 2015; Wasson, 2019). Bowling (2005, p. 343)
points out that “It has been proposed that concepts such as health status, QoL and
HRQoL [health related QoL], when used as outcome variables, are more appropriately
measured with a global single item. This is because multi-domain measures confound
the dimensionality of these concepts with the multiplicity of their causal sources.”
Using a single measure was additionally important for capturing the fact that “QOL
can also be negative” (Kemp, 1999, p. 159); and thus for exploring how low and high QoL scores
correlated with other variables.

Translating the study questionnaire into the relevant local languages
(Bosnian/Croatian/Serbian, Spanish, and the Acholi and Lango dialects of the Luo
language) was a crucial part of the research process. Van Ommeren et al. (1999) note that “One
sequence, popular in the field, has been developed by Brislin (1976, p. 287). He suggested a
five-step translation process: (a) translation; (b) blind back-translation; (c)
examination of original, translation and blind back-translation; (d) pilot study;
and (e) examination of pilot study data and subjects.” We similarly adopted this
process. It is important to note that we were not simply aiming for what (Peña, 2007, p. 1256) has
termed linguistic equivalence, meaning the very literal translation of English words
into the local languages. This would have been too blunt, particularly in the case
of Uganda. Discussing the challenges of research translation from English to Luo,
and using the example of compound words such as firewood, sawdust, and household,
Omona and Groce (2021) note that “Some of them had meanings that had nothing to do with
the individual words involved.” The Luo language also uses many metaphors. During
the piloting of the questionnaire in Uganda, for example, one of the
participants—referring to his desire to have some psychological support—talked about
“putting a warm cloth on the wounded place.” In the case of all three countries,
thus, a key aim was to ensure that the translation made sense in the particular
cultural context. In this regard, (Peña, 2007, p. 1258) talks about “cultural
equivalence” that “focuses more centrally on the way members of different cultural
and linguistic groups view or interpret the underlying meaning of an item.” It is
for this reason that multiple people (including from some of the in-country
organizations) were involved in the translation process, in addition to professional
translators.

Validation of the questionnaire occurred in two ways. First, it was shared with the
aforementioned in-country organizations involved in the study, and they were invited
to comment, *inter alia*, on the wording and ordering of the
questions, the scales used, and anything that they felt was problematic or needed to
be changed, including irrelevant items. Second, the questionnaire was piloted during
research team visits to each country between January and April 2018. A total of 32
female and male victims-/survivors (11 in BiH, 10 in Colombia and 11 in Uganda) took
part in the pilots, which were extremely useful in highlighting issues that needed
to be addressed.

For example, two particular problems with the TEC emerged during the piloting. First,
the use of the word “witnessed” (e.g., “Witnessed the destruction of your home or
other people’s home”) caused some confusion; some participants interpreted it as
meaning that they had “witnessed” in the sense of giving testimony in court. Second,
the question “*Other than* the situations described above, has
anything else ever happened to you that was very frightening, dangerous, or
violent?” frequently elicited a long narrative (which could have been anticipated).
For this reason, the question was removed from the post-pilot version of the
questionnaire. Including it in the pilot version, however, was an important
opportunity to ascertain whether the TEC items covered the full range of experiences
that participants found distressing.

During the piloting process, it also quickly became apparent that parts of the CES,
and especially the statement “This event has become a reference point for the way I
understand myself and the world,” were not easily understandable to some of the
participants. To address this, additional explanations were prepared for six of the
seven statements in the scale (one of the statements did not require any further
clarification). This was a way of ensuring that all participants would receive the
same explanation if they did not understand a particular statement. These additional
explanations were very effective and participants did not have any difficulties
understanding them. For the above-mentioned statement, for example, the additional
explanation used was “To explain myself and the world around me, I always refer back
to the sexual violence I experienced.” Those who participated in the piloting of the
questionnaire were also asked for their views on it, whether they would add or
change anything and whether they found any parts of it difficult.

The first author, two postdoctoral researchers and the aforementioned in-country
organizations applied both the pilot questionnaire and the final questionnaire. Two
independent psychologists with experience of working with victims-/survivors of
conflict-related sexual violence, in BiH and Colombia respectively, also conducted a
small number of administrations. The sensitivity of the subject matter, the fact
that some of the participants were illiterate and the need to mitigate the risk of
possible low response rates meant that the questionnaires were read aloud rather
than self-administered.

### Analyses

The first aim of the study was to use CFA to gauge the conceptual and measurement
equivalence of the ARM across sites. However, the traditional factor structure
of the model resulted in a poor fit across the sites and led to the need to
conduct exploratory factor analyses (EFA) for each one, to determine new factor
structures. For further detail regarding the CFA and the use of the EFAs, see
Appendix A.

Following identification of appropriate models for each country, we used
Mann-Whitney U-tests and one-way analysis of variance (ANOVA; Kruskal-Wallis) to
examine how individuals within each country compared in terms of their ARM
factor scores based on sociodemographic and diversity variables, including their
age, ethnicity, and education level. Given that male participants reflected only
a small proportion of the total sample (for reasons discussed in the previous
section), we repeated analyses with just the female participants and the results
did not significantly change. Male participants were accordingly left in, both
for diversity reasons and to retain a suitable level of power for the
analyses.

We also compared scores on the emergent ARM factors with the key psychosocial
variables measured in the questionnaire. Effect sizes (epsilon squared and
Cohen’s *d*) are reported for all significant results and
interpreted using thresholds (Cohen, 1992, 2013). All analyses were undertaken
using *Jamovi* v1.6.3.0 (The Jamovi Project, 2020).

## Results

### BiH

In the BiH sample, the scree plot generated as part of the EFA indicated that
models consisting of three to five factors were potentially suitable. Of these
options, a four-factor model emerged as the most well-fitting solution (RMSEA =
.06, [90% CI = .05-.08]) and the latent factors correlated appropriately. The
items in the four-factor model reflected individual differences in:
*factor 1: Social and community relations*; *factor 2:
Family support and relationships*; *factor 3: Cultural
participation and belonging*, and *factor 4: Abilities and
opportunities* (Table B.2). Each of the items appeared to load
distinctly onto a factor, aside from items 16, 25, and 27, which cross-loaded
onto multiple factors. Items 16 and 27 loaded more strongly onto a single factor
and so were not permitted to cross-load. However, given that item 25 (“I have
opportunities to apply abilities in life”) loaded similarly onto factors 3 and
4, which may reflect an important interaction of individual engagement and
contexts that provide opportunities, it was permitted to remain cross-loading on
both factors.

As the distribution of scores within the factors was slightly negatively skewed
(which is common for the measure; e.g., Borualogo & Jefferies, 2019),
nonparametric tests were used to investigate potential differences between
sociodemographic groups in terms of their factor scores. However, no significant
differences were determined (Table B.3).

Associations between the emergent ARM factors and the psychosocial variables were
examined (Table B.4). *Social and community relations (factor 1)*
positively correlated with feelings of safety in the community and feeling able
to ask for help (marginally with perceived QoL, *p* = .048).
*Family support and relationships (factor 2)* negatively
correlated with consequences of sexual violence and number of current problems,
and positively correlated with feelings of safety in the community, feeling able
to ask for help, and QoL (marginally with perceived health, *p* =
.040). Interestingly, *cultural participation and belonging (factor
3)* was positively associated with traumatic events, feeling safe in
the community, and feeling able to ask for help, but negatively correlated with
current problems. Finally, *abilities and opportunities (factor
4)* positively correlated with feeling safe in the community and
feeling able to ask for help.

## Colombia

In the Colombian sample, the scree plot indicated that models consisting of three to
six factors could be appropriate, but a four-factor model was the best-fitting model
(RMSEA = .06, [90% C I= .05-.07]) and the factors also correlated appropriately. The
items in this four-factor model appeared to cluster into the following:
*factor 1: Family support and relationships*; *factor 2:
Community support and belonging*; *factor 3: Contextual support
and opportunities*; *factor 4: Support from friends*
(Table B.5). Three items cross-loaded onto multiple factors (items 12, 21, and 26).
Given the similarities in the magnitude of the loadings, as well as making
contextual sense, the items were allowed to cross-load.

Scores on the emergent factors in the Colombian sample did not generally differ
across the sociodemographic variables (Table B.6). That said, there was a modest but
significant difference in *family support and relationships*
according to level of education (*p* = .046,
*ε*^2^ = .05). Specifically, pairwise comparisons
indicated that those who had completed technical college (*técnica
professional*) had significantly higher scores on the factor
(*M* = 26.19, *SD* = 5.88) than those with no
schooling (*M* = 21.05, *SD* = 6.77). There was a
similarly modest difference in the *contextual support and
opportunities* factor (*p* = .022,
*ε*^2^ = .06) by education level, with higher scores for
those with secondary school (*M* = 43.83, *SD* = 4.65)
compared to those who had only completed primary school education
(*M* = 41.26, *SD* = 5.85).

For both *community support and belonging* and *contextual
support and opportunities*, participants who lived in cities reported
significantly higher scores (*M* = 38.96, *SD* = 6.78;
*M* = 43.93, *SD* = 3.61, respectively) compared
to those in rural environments (*M* = 35.18, *SD* =
7.72; *M* = 40.31, *SD* = 6.80, respectively).
Finally, employed participants reported significantly higher levels of
*community support and belonging (M* = 35.51, *SD*
= 7.76) and *support from friends* factors (*M* =
5.64, *SD* = 2.52, *d* =.22) than unemployed
participants (*M* = 38.44, *SD* = 7.43,
*M* = 6.60, *SD* = 2.49, respectively). Though
modest (*d* = .22 and *d* = .22 respectively), these
differences suggest that employment constitutes an important resource for managing
economic stressors.

Individual differences in *family support and relationships* were
negatively associated with current problems and positively associated with perceived
health and QoL (Table B.7). *Community support and belonging*
correlated positively not only with perceived health and QoL, but also with feeling
safe in the community and feeling able to ask for help. *Contextual support
and opportunities* was associated with feeling able to ask for help
(*p* = .001), and, interestingly, *support from
friends* was positively associated with CES scores.

## Uganda

In the Uganda sample, the scree plot indicated that models consisting of three to six
factors could be appropriate. Model fit estimates indicated that a six-factor model
was the most appropriate solution (RMSEA = .05, [90% CI = .04-.07]) and the factors
correlated appropriately. The items in this six-factor model reflected:
*factor 1: Cultural and social bonds*; *factor 2: Familial
bonds*; *factor 3: Individual strengths*; *factor
4: Cooperation and community*; *factor 5: Relationships with
friends and community*; and *factor 6: Family resources and
support* (Table B.8). Item 23 (“I think it is important to support my
community”) cross-loaded onto factors 1 and 5, which is in line with their community
nature.

There were modest but significant differences in *individual
strengths* depending on the individual’s ethnicity (*d* =
.28), with Acholi participants scoring higher (*M* = 20.07,
*SD* = 3.81) than participants who identified as Lango
(*M* = 18.47, *SD* = 2.94). However, the reverse
was found for *relationships with friends and community*
(*d* = .36); Lango participants scored significantly higher
(*M* = 15.72, *SD* = 2.73) than those identifying
as Acholi (*M* = 13.65, *SD* = 3.73; Table B.9).

Modest differences were found for *familial bonds* according to
marital status (*d* = .28), with married participants having higher
scores (*M* = 12.95, *SD* = 2.25) than unmarried
participants (*M* = 11.44, *SD* = 3.14). There were
also modest differences in *cultural and social bonds*
(*d* = .25) and *relationships with friends and
community* (*d* = .21) according to family size.
Individuals with fewer than four children had higher scores (*M* =
27.08, *SD* = 2.79, *M* = 15.44, *SD* =
3.13 respectively) than those with four or more children (*M* =
25.55, *SD* = 3.86, *M* = 15.44, *SD* =
3.13, respectively).

Finally, modest but significant urban–rural contrasts were observed. For
*individual strengths*, this difference (*p* =
.034, *ε*^2^ = .05) indicated that individuals in cities had
significantly higher scores (*M* = 20.61, *SD* = 3.62)
than those in villages (*M* = 18.94, *SD* = 3.27). The
reverse was true for *relationships with friends and community*
(*ε*^2^ = .09) and *family resources and
support* (*ε*^2^ = .11), with participants in
cities having lower scores (*M* = 12.79, *SD* = 3.81;
*M* = 7.88, *SD* = 2.86, respectively) than those
in trading centers (*M* = 15.04, *SD* = 3.23;
*M* = 10.26, *SD* = 2.98, respectively) and
villages (*M* = 15.29, *SD* = 3.09; *M*
= 10.19, *SD* = 2.65, respectively).

In terms of the psychosocial variables, both *cultural and social
bonds* and *relationships with friends and community*
positively correlated with CES scores. *Relationships with friends and
community, family resources and support,* and *familial
bonds* positively correlated with feeling able to ask for help.
*Familial bonds* were also negatively associated with current
problems, and positively associated with perceived QoL. *Individual
strengths* were negatively correlated with consequences of sexual
violence and current problems, but were positively associated with feeling safe in
the community, perceived health and QoL. *Cooperation and community*
scores positively correlated with feeling safe in the community (Table B.10).

## Discussion

Our findings demonstrate that there can be significant differences—as well as some
broad commonalities—on a measure of adult resilience between countries where
populations share similar experiences of violence but come from very different
cultures. In BiH, notwithstanding the legacy of the 1992-1995 war and the continuing
constitutional division of the country along ethnic lines, the factor structure of
the ARM reveals that *social and community relations* (factor 1)
constitute a significant protective resource. It is interesting to note in this
regard the positive correlation between *social and community
relations* and feelings of safety, particularly as many of the
participants were living in ethnically-mixed areas. That *social and
community relations* also positively correlated with feeling able to ask
for help and perceived QoL suggests that, at least in some areas, a multi-ethnic way
of life persists (O’Loughlin, 2010). *Cultural participation and belonging* (factor 3),
similarly, point to a deeper level of “resilience” within the sub-strata of Bosnian
communities. In particular, factor 3’s positive correlations with TEC scores,
feeling safe in the community, and feeling able to ask for help indicate the
protective functioning of sociocultural dynamics.

Community was also very important in Colombia, but in a different way. Some of the
participants were social leaders who, because of their activism, had faced death
threats. That *community support and belonging* (factor 2) correlated
with feeling safe in the community suggests that the work that these women did,
notwithstanding the dangers (Reuters, 2020), was a protective factor in their lives in the sense of
giving them a purpose. Further highlighting this, *contextual support and
opportunities* (factor 3) enabled participants to ask for help, and in
some cases they were asking not only for themselves but also for those who were part
of their organizations. Higher scores with respect to both *community support
and belonging* and *contextual support and opportunities*
among participants in urban areas were unsurprising given that rural areas “have
historically borne the brunt of the armed conflict in Colombia” (Rosas, 2018), in part due
to weak state control.

In Uganda, the fact that three of the factors had a community
dimension—*cultural and social bonds* (factor 1),
*cooperation and community* (factor 4) and *relationships
with friends and community* (factor 5)—attests to the fundamental
significance of community in participants’ lives. Community was particularly
important in providing a sense of safety, which is noteworthy given that some of the
participants were living in border areas that continued to experience violent cattle
raids from the Karamojong, a pastoralist group in northeast Uganda. The
significantly higher scores among Lango participants compared to Acholi participants
with respect to *relationships with friends and community* may be
explained by the nature of the war in northern Uganda, which began in Acholiland and
brutally tore apart families and communities, “[*w*]ith nearly the
entire rural population of Acholiland displaced into internment camps” (Branch, 2007, p. 194).

The overall findings additionally point to the significance—and resilience—of
families. Nelson (2003,
p. 312) notes that “The role of the family in a traumatized society can be both a
sanctuary of safety and protection for its members and an area of pain and
destruction that parallels the horrors of the larger society that are projected onto
the family system.” While the results from the TEC showed that participants’
distressing experiences frequently included their families (e.g., seeing a loved one
being beaten), *family support and relationships* (factor 2) emerged
as a key protective factor in BiH, further highlighted by its correlation with other
items in the questionnaire, including QoL.

In Colombia, *family support and relationships* (factor 1) correlated
negatively with current problems and positively with perceived health and QoL,
suggesting that family was also an important protective resource within Colombian
participants’ lives. The loading of item 12 (“I talk to my family/partner about how
I feel”) onto factors 1 and 2, however, was consistent with the fact that during the
application of the questionnaire, some of the participants raised questions about
the meaning of “family,” which underscores the fluidity of the concept (Parry, 2005). Moreover,
the long duration of the armed conflict in Colombia had taken a significant toll on
families (although this was the case in all three countries). During the application
of the aforementioned TEC, for example, 90 Colombian respondents said that they had
experienced family members being “disappeared” (most commonly by paramilitary
groups) and 113 said that members of their family had been killed. Potentially,
therefore, the boundaries of “family” and “community” have somewhat blurred, with
communities essentially stepping in and playing the role of a “family.” The
cross-loading of item 26 (“I enjoy my family/partner’s cultural and family
traditions”) onto factors 1 and 2 further supports this.

It is also noteworthy regarding Colombia that two of the items loaded to form a
factor specifically about *support from friends* (factor 4). Because
many of the Colombian participants were internally displaced, and were separated
from or had lost their families, friends—including other victims-/survivors of
conflict-related sexual violence—were an important protective resource for some of
them. *Support from friends* positively correlated with CES scores,
suggesting that the centrality of the sexual violence in participants’ lives created
a need for them to turn to others (often in the context of women’s associations) who
had gone through similar experiences.

That the Ugandan model has the largest number of factors partly reflects the
complexity of social relationships, particularly evinced through notions of
kinship—defined as “the social organization and cultural meanings of relatedness
through descent and through marriage (affinity)” (Peters, 2019, p. 34). The fact that
participants who were married scored slightly higher on *familial
bonds* (factor 2) illustrates the importance of kinship. The existence
of two family-related factors, *familial bonds* and *family
resources and support* (factor 6), further points to the rich meanings
and complex functioning of family in this context.

In BiH, the smallest number of items loaded onto *abilities and
opportunities* (factor 4). This was unsurprising. More than 20 years
after the war in BiH ended, a strong sense of apathy has set in, fueled by a
perceived absence of significant change and progress, particularly vis-à-vis the
political and economic situation (Bennoni & Ramović, 2020, p. 47). Alongside a
macro level narrative of stagnation, however, positive correlations with feeling
safe in the community and feeling able to ask for help point to important “movement”
at the local level.

Indeed, this idea of movement emerges from all of the factor models in different
ways, in the sense of drawing attention to the enabling dynamics of protective
factors. This, in turn, illuminates a larger point. Policy discussions about
conflict-related sexual violence often embrace a “deficit model” (Burstow, 2003, p. 1311),
through a focus on victims-/survivors’ unmet needs, including for psychosocial
support and healthcare. At the international level, the widespread emphasis on a
“survivor-centered approach” to dealing with conflict-related sexual violence—a
concept formally adopted by the United Nations Security Council (2019) as part of its Women, Peace and
Security agenda—is fundamentally about putting the needs and priorities of
victims-/survivors first (International Committee of the Red Cross, 2020).

Frequently overlooked within such discussions about conflict-related sexual violence,
however, are the resources that victims-/survivors have in their daily lives. This
research, because of its particular focus on resilience, offers something new in
this regard. The different factor structures for each country bring to the fore
diverse and varied clustering of protective resources. What they underscore, thus,
is the need for policy interventions, including transitional justice interventions
(Clark & Ungar, 2021), that not only address resource deficits but also, as a
complementary approach, give attention to and invest in victims-/survivors’ resource
structures, including family and community. The crucial point in this regard is that
“centring” those who have suffered conflict-related sexual violence also means
bringing to the forefront the social ecologies with which their lives are
inextricably intertwined (Clark, 2021a).

## Limitations

One of the main limitations of the study is sample size. While the total sample size
was 449, we decided, based on the results of the CFA, that it made most sense to
proceed on a country-by-country basis as opposed to combining the three datasets.
There are divergent views of what constitutes an acceptable minimum sample size for
EFA, but Worthington and Whittaker (2006, p. 817) point out that “In general, there is some
agreement that larger sample sizes are likely to result in more stable correlations
among variables and will result in greater replicability of EFA outcomes.” They also
suggest that a minimum of 300 participants constitutes an adequate sample size
(Worthington & Whittaker, 2006, p. 817). Nevertheless, some studies using EFA have had
significantly fewer participants; Liebenberg and Moore’s (2006) study of survivors
of Irish clerical institutional abuse, for example, included 105 participants. De Winter et al. (2009, p.
149), moreover, note that “it remains undefined how small a sample size can be and
still yield acceptable solutions,” also underlining the important point that “EFA is
indeterminate by nature, but so is the empirical world” (De Winter et al., 2009, p. 178). The
larger point is that the relatively small size of each of the country samples means
that the models are necessarily tentative. CFA would therefore be useful to validate
each country model.

Particularly from a diversity perspective, a significant limitation is the small
number of male participants. Certainly, the results cannot be automatically
generalized to male participants at this time, but this would be an interesting
avenue for further research.

A further limitation is that the scores for the TEC, CES, and current problems were
based on summed totals. While this is not unusual (see, e.g., Heathcote & Simons, 2020), each item
in these scales is a distinct issue that may affect people or relate to the factors
in distinct ways. Hence, they are not necessarily equally interchangeable indicators
of the variable in question, like trauma. This is one of the issues with such
measures and future research could disaggregate them, for example by looking at the
impact of specific types of potentially traumatic events or particular types of
problems.

Finally, because the dataset was cross-sectional, it was not possible to ascertain
the direction of effects. For example, does having stronger familial bonds lead to
fewer current problems, or does having fewer current problems allow people to focus
on nurturing familial bonds, or does it work both ways? The fact that the data form
part of a broader mixed methods study means that the qualitative data can partly
help to address such issues (see, e.g., Clark, 2021a). Additionally, longitudinal
research could add further clarity (see, e.g., Weziak-Bialowolska et al., 2020).

## Conclusion

Notwithstanding an expansive body of literature on resilience and a similarly large
body of scholarship addressing the issue of conflict-related sexual violence,
neither one to date has given much attention to the other. In seeking to address
this gap, this research is the first to apply the ARM to victims-/survivors of
conflict-related sexual violence. Based on both CFA and EFA, it has ultimately
generated three different factor structures for BiH, Colombia, and Uganda,
suggesting that a single factor structure does not sufficiently capture the diverse
clustering of protective factors linked to the particularities of each country,
including the dynamics of the conflicts themselves. While the sample size
underscores the importance of further research, the results strongly support the
need—particularly at the policy level—for greater attention to be given to
victims-/survivors’ social ecologies and to the potential resources that they
offer.

This, in turn, resonates with broader scholarship on resilience—and in particular
social-ecological systems. This concept, referred to in the introduction,
specifically recognizes and emphasizes interconnections between people and
ecological systems (see, e.g., Folke et al., 2005, pp. 443-444; Cinner & Barnes, 2019, p. 51). In the
context of conflict-related sexual violence, the relevance of the concept is that it
provides a framework for thinking about the different ways that individuals’
ecologies can support and foster social processes that help victims-/survivors to
rebuild and move on with their lives.

## Appendix A: Confirmatory and Exploratory Factor Analyses

## Confirmatory Factor Analysis (CFA)

Given that other studies employing the ARM have found varying factor structures
(e.g., Arslan, 2015;
Liebenberg & Moore, 2018), our first aim was to use CFA to assess the conceptual and
measurement equivalence of the ARM factor structure across sites. To evaluate the
CFA, we used a maximum likelihood estimator and evaluated model fit using the
established criteria of a comparative fit index (CFI) and Tucker-Lewis Index (TLI)
>.90 (Hu & Bentler, 1999) and a root mean square error of approximation (RMSEA) and
standardized root mean square residual (SRMR) <.08 (Hu & Bentler, 1999).

An initial CFA applied to the entire dataset resulted in poor fit (CFI = .69, TLI =
.66, RMSEA = .08 [90% = .07 -.08], SRMR = .08). When checking the fit per country,
similar poor fit statistics were observed: BiH: CFI = .70, TLI = .67, RMSEA = .09,
[90% CI = .08 -.10], SRMR = .10; Colombia: CFI = .69, TLI = .66, RMSEA = .09, [90%
CI = .08 -.09], SRMR = .08; Uganda: CFI = .57, TLI = .53, RMSEA = .09, [90% CI = .08
-.10], SRMR = .09). Although reviewing the modification indices suggested some ways
in which the model could be improved (by freeing parameters), these improvements
still did not result in a model with adequate fit, suggesting the original
three-factor structure of the ARM should be reconsidered.

## Exploratory Factor Analysis (EFA)

We accordingly revisited the factor structure of the ARM through EFA to determine a
better-fitting model. We chose to use EFA (rather than principal component analysis)
to identify the underlying dimensions of the measure (for other examples of EFAs
applied to the CYRM/ARM, see Robinson et al., 2016; Amini-Tehrani et al., 2020; Kaunda-Khangamwa et al., 2020). While similar to principal component analysis (PCA), EFA is widely
considered as the appropriate approach when investigating the dimensionality of
social and psychological constructs because, unlike PCA, it takes account of
measurement error and shared variance (Brown, 2006).

Given the variation in the CFA fit statistics for each country sample, and the
variation in factor structures when the ARM has been used in other countries (e.g.,
see Van Rensburg et al. 2017; Liebenberg & Moore, 2018), we determined that individual EFAs for each country
would result in the most contextually appropriate solutions. For each country
sample, Barlett’s Test of Sphericity produced a significant finding
(*p* <.001), indicating interrelationships between the
variables (Field, 2009),
and a Kaiser-Meyer-Olkin test for sampling adequacy confirmed that values fell
between .6 and 1.0 (Tabachnick & Fidell, 2006) (BiH = .77; Colombia = .77; Uganda = .73).

For the EFAs, we used a maximum likelihood extraction technique and an oblique
rotation strategy (oblimin), given that others have found highly correlated factors
in previous structural investigations of the CYRM and ARM (e.g., Liebenberg et al., 2012).
To determine factor structure we used Comrey and Lee’s generally accepted thresholds
for item loading values, where items loading ≥ .32 are considered the minimum values
for loading. Items that cross-load (loadings ≥ .32 on two or more factors) can be
managed in various ways (see Yong & Pearce, 2013). Some suggest that a minimum separation between
factor loadings indicates how to manage an item (Matsunaga, 2010; Howard, 2016), while others retain
cross-loading items regardless (e.g., Le & Cheong, 2010). We reviewed each
cross-loading item to see if the loading separation suggested that an item could be
dropped from a particular factor. However, we were also open to retaining
cross-loading items, given that some of the items in the ARM were likely to relate
to multiple dimensions of resilience.

We then used multiple criteria to assess and select an appropriate model; including
examining scree plots and eigenvalues, RMSEA values <.08 (Hu & Bentler, 1999), ensuring factors
correlated appropriately and also Henson and Roberts’ (2006, p. 399)
“reasoned reflection” concerning sensible configurations of the items per factor in
factor loading matrices. In sum, we sought a parsimonious model for each country
that had good statistical properties and one that possessed relatively clear and
distinct factors.

## References

Amini-TehraniM., NasiriM., SadeghiR., HoseiniE. -S., JalaliT., & ZamanianH. (2020).
Social-ecological measure of resilience: An adapted
measure for Persian-speaking university students.
*Health Promotion Perspectives*,
10(3),
207-219.
https://doi.org/10.34172/hpp.2020.xx3280275710.34172/hpp.2020.34PMC7420169ArslanG. (2015). Psychometric
properties of adult resilience measure (ARM): The study of reliability
and validity. *Ege Eğitim
Dergisi*, 16(2),
344-357.BrownT. A. (2006). *Confirmatory
factor analysis for applied research*.
Guilford Press.FieldA. (2009). *Discovering
statistics using SPSS*. (3rd ed.).
SAGE Publications.HensonR. K., & RobertsJ. K. (2006). Use of
exploratory factor analysis in published research: Common errors and
some comment on improved practice.
*Educational and Psychological
Measurement*, 66(3),
393-416.
https://doi.org/10.1177/0013164405282485HowardM. C. (2016). A review of
exploratory factor analysis decisions and overview of current practices:
What we are doing and how can we improve?
*International Journal of Human–Computer
Interaction*, 32(1),
51-62.
https://doi.org/10.1080/10447318.2015.1087664HuL., & BentlerP. M. (1999). Cutoff
criteria for fit indexes in covariance structure analysis: Conventional
criteria versus new alternatives.
*Structural Equation Modeling: A Multidisciplinary
Journal*, 6(1),
1-55.
https://doi.org/10.1080/10705519909540118Kaunda-KhangamwaB. N., MaposaI., DambeR., MalisitaK., MtagalumeE., ChigaruL., MunthaliA., ChipetaE., PhiriS., & MandersonL. (2020). Validating a
child youth resilience measurement (CYRM-28) for adolescents living with
HIV (ALHIV) in urban Malawi. *Frontiers
in Psychology*, 11.
https://doi.org/10.3389/fpsyg.2020.0189610.3389/fpsyg.2020.01896PMC748820832982826LeT. C., & CheongF. (2010). Perceptions
of risk and risk management in Vietnamese catfish farming: An empirical
study. *Aquaculture Economics &
Management*, 14(4),
282-314.
https://doi.org/10.1080/13657305.2010.526019LiebenbergL., & MooreJ. C. (2018). A social
ecological measure of resilience for adults: The
RRC-ARM. *Social Indicators
Research*, 136,
1–19.
https://doi.org/10.1007/s11205-016-1523-yLiebenbergL., UngarM., & van de VijverF. (2012). Validation of
the child and youth resilience measure-28 (CYRM-28) among Canadian
youth. *Research on Social Work
Practice*, 22(2),
219-226.
https://doi.org/10.1177/1049731511428619MatsunagaM. (2010). How to
factor-analyze your data right: Do’s, don’ts, and
how-to’s. *International Journal of
Psychological Research*,
3(1),
97-110.
https://doi.org/10.21500/20112084.854RobinsonR. V., DavidE. J. R., & HillM. (2016). Participatory
mixed methods research across cultures. In JasonL. A. & GlenwickD. S. (Eds.), *Handbook of
methodological approaches to community-based research: Qualitative,
quantitative, and mixed methods* (pp.
273-282). Oxford
University Press.TabachnickB. G., & FidellL. S. (2006). *Using
multivariate statistics* (5th ed.).
Allyn & Bacon.Van RensburgA. C., TheronL. C., & UngarM. (2017). Using the
CYRM-28 with South African young people: A factor structure
analysis. *Research on Social Work
Practice*, 29(1),
1-10.
https://doi.org/10.1177/1049731517710326YongA. G., & PearceS. (2013). A beginner’s
guide to factor analysis: Focusing on exploratory factor
analysis. *Tutorials in Quantitative
Methods for Psychology*,
9(2),
79-94.

## Appendix B: Tables

**Table B1. table1-08862605211028323:** Respondents (*n* = 449) by Ethnicity.

**BiH**	**Colombia**	**Uganda**
Bosniak *n* = 85	Afro-Colombian *n* = 49	Acholi *n* = 76
Serb *n* = 30	Mestizo *n* = 44	Lango *n* = 76
Croat *n* = 6	Indigenous *n* = 19	
Other *n* = 5	Other *n* = 47	
	Did not understand *n* = 12

**Table B2. table2-08862605211028323:** Factor Loadings of the Four-factor Model for BiH.

	**1. Social and Community Relations**	**2. Family Support and Relationships**	**3. Cultural Participation and Belonging**	**4. Abilities and Opportunities**
Item 18	**.73**			
Item 19	**.71**			
Item 16	**.61**		.33	
Item 15	**.60**			
Item 14	**.55**			
Item 21	**.50**			
Item 23	**.41**			
Item 11	**.34**			
Item 2				
Item 17		**.91**		
Item 5		**.88**		
Item 24		**.76**		
Item 6		**.34**		
Item 12		**.32**		
Item 3				
Item 26			**.80**	
Item 27	.36		**.72**	
Item 28			**.46**	
Item 22			**.45**	
Item 25			**.44**	**.34**
Item 10			**.40**	
Item 9				
Item 4				**.60**
Item 13				**.56**
Item 8				**.41**
Item 7				
Item 20				
Item 1				

*Note*. Items in bold were retained on the factor.

**Table B3. table3-08862605211028323:** Descriptive Statistics (Mean, SD) for the Factors and Group
Comparisons in the BiH Sample.

	1. Social and Community Relations	**2. Family Support and Relationships**	**3. Cultural Participation and Belonging**	**4. Abilities and Opportunities**
Overall sample	30.34 (5.95)	20.44 (4.14)	24.36 (4.21)	16.83 (2.59)
*Age (median split)*				
<55 (n = 58)	29.58 (6.43)	20.26 (4.40)	24.23 (4.15)	17.05 (2.50)
≥55 (n = 68)	30.99 (5.49)	20.60 (3.93)	24.47 (4.29)	16.63 (2.66)
Mann-Whitney U test	*p* = .315	*p* = .935	*p* = .661	*p* = .376
*Ethnicity* ‖				
Bosniak (n = 84)	29.93 (6.28)	20.13 (4.42)	24.58 (6.06)	16.58 (2.39)
Serbian (n = 30)	31.67 (4.97)	21.50 (3.17)	24.33 (3.34)	17.33 (2.02)
Mann-Whitney U test	*p* = .302	*p* = .208	*p* = .491	*p* = .295
*Marital status*				
Not married (n = 24)	29.96 (4.95)	20.00 (4.29)	23.29 (5.55)	16.33 (2.84)
Married (n = 65)	30.37 (5.72)	20.42 (3.96)	24.16 (3.94)	16.98 (2.42)
Mann-Whitney U test	*p* = .694	*p* = .714	*p* = .796	*p* = .375
*Number of children* †				
None (n = 25)	32.00 (4.90)	19.56 (5.29)	23.32 (5.44)	16.04 (2.73)
1 (n = 20)	29.80 (6.67)	21.00 (3.87)	24.80 (3.62)	17.15 (2.28)
2+ (n = 81)	29.99 (6.04)	20.58 (3.80)	24.57 (3.90)	16.99 (2.60)
One-way ANOVA	*p* = .299	*p* = .692	*p* = .687	*p* = .235
*Education* ‡				
Primary school (n = 58)	30.48 (9.76)	19.83 (4.40)	24.74 (4.29)	16.86 (2.66)
Secondary school (n = 51)	29.96 (5.13)	21.20 (3.46)	23.41 (4.28)	16.65 (2.53)
Mann-Whitney U test	*p* = .462	*p* = .127	*p* = .072	*p* = .559
*Location* §				
Town (n = 44)	30.43 (5.41)	19.66 (4.70)	23.20 (4.35)	16.59 (.264)
Suburbs (n = 44)	30.00 (5.79)	20.50 (3.45)	24.74 (4.57)	16.80 (2.81)
Village (n = 33)	30.06 (6.83)	21.03 (4.33)	25.15 (3.55)	17.06 (2.33)
One-way ANOVA	*p* = .967	*p* = .272	*p* = .050	*p* = .760
*Employment status*				
Unemployed (n = 91)	30.23 (6.07)	20.31 (4.07)	24.43 (4.35)	17.00 (2.78)
Employed (n = 25)	30.76 (5.00)	21.28 (4.27)	24.20 (4.02)	18.00 (1.87)
Mann-Whitney U test	*p* = .833	*p* = .146	*p* = .703	*p* = .233

*Note*. ANOVA uses Kruskal-Wallis test; † Groups were
created using a median split and a “no children” group; ‡ Only a small
number of participants completed university or did not complete primary
school. § Only five participants reported living in a city, so were
excluded from the comparative analysis. ‖ Six individuals identified as
Croat and five as “other,” but these groups were small and so excluded
from the comparative analysis.

**Table B4. table4-08862605211028323:** Correlations Between the ARM Factors and Psychosocial Variables in
the BiH Sample.

	**1. Social and Community Relations**	**2. Family Support and Relationships**	**3. Cultural Participation and Belonging**	**4. Abilities and Opportunities**
1. TEC	.08	.02	.24**	.10
2. CES	.06	.00	.17	.05
3. Consequences of sexual violence	-.14	-.22*	-.21*	-.10
4. Current problems	-.17	-.23**	-.22*	-.08
5. Feeling safe in community	.31***	.32***	.32***	.30***
6. Feeling able to ask for help	.40***	.39***	.31***	.25**
7. Perceived health	.11	.18*	-.01	.04
8. Perceived QoL	.18*	.28**	.07	.07

*Note*. All correlations are Spearman; **p*
< .05, ***p* < .01, ****p* <
.001.

**Table B5. table5-08862605211028323:** Factor Loadings of the Four-factor Model For Colombia.

	**1. Family Support and Relationships**	**2. Community Support and Belonging**	**3. Contextual Support and Opportunities**	**4. Support From Friends**
Item 17	**.81**			
Item 5	**.79**			
Item 6	**.73**			
Item 24	**.65**			
Item 12	**.47**	**.34**		
Item 7	**.35**			
Item 27		**.61**		
Item 25		**.55**		
Item 15		**.49**		
Item 16		**.46**		
Item 26	**.35**	**.44**		
Item 23		**.40**		
Item 20		**.38**		
Item 19		**.36**		
Item 21		**.35**	**.33**	
Item 22				
Item 28				
Item 4			**.61**	
Item 1			**.57**	
Item 3			**.50**	
Item 11			**.45**	
Item 9			**.45**	
Item 2			**.40**	
Item 10			**.39**	
Item 8			**.35**	
Item 13			**.32**	
Item 14				**1.01**
Item 18				**.71**

*Note*. Items in bold were retained on the factor.

**Table B6. table6-08862605211028323:** Descriptive Statistics (Mean, SD) for the Factors and Group
Comparisons in the Colombian Sample.

	**1. Family Support and Relationships**	**2. Community Support and Belonging**	**3. Contextual Support and Opportunities**	**4. Support From Friends**
Overall sample	24.16 (6.65)	37.24 (7.18)	42.53 (5.26)	6.32 (2.43)
*Age (median split)*				
<42 (n = 79)	28.34 (5.45)	17.13 (5.39)	23.00 (4.04)	16.10 (2.73)
≥42 (n = 91)	30.02 (6.59)	16.76 (5.43)	24.08 (3.86)	16.52 (2.70)
Mann-Whitney U test	*p* = .057	*p* = .679	*p* = .137	*p* = .289
*Ethnicity*				
Afro-Colombian (n = 49)	24.61 (6.22)	37.24 (7.31)	42.90 (5.04)	5.90 (2.50)
Indigenous (n = 19)	23.74 (6.33)	35.89 (6.21)	41.50 (6.56)	6.63 (1.71)
Mestizo (n = 44)	24.98 (6.70)	37.98 (8.43)	41.61 (6.21)	6.50 (2.57)
‘Other’ (n = 47)	23.62 (7.04)	37.36 (6.22)	43.15 (3.83)	6.45 (2.49)
One-way ANOVA	*p* = .769	*p* = .748	*p* = .477	*p* = .517
*Marital status*				
Not married (n = 65)	23.17 (7.00)	35.98 (6.94)	42.52 (4.87)	6.11 (2.59)
Married (n = 21)	23.75 (6.48)	35.26 (9.66)	42.21 (5.18)	6.29 (2.37)
Mann-Whitney U test	*p* = .733	*p* = .764	*p* = .817	*p* = .772
*Number of children* †				
None (n = 13)	22.38 (7.07)	34.92 (7.58)	41.31 (3.88)	5.23 (2.31)
1-2 (n = 49)	23.53 (7.88)	36.94 (7.58)	42.32 (5.02)	6.24 (2.45)
3+ (n = 108)	24.65 (5.99)	37.59 (7.02)	42.74 (5.54)	6.44 (2.31)
One-way ANOVA	*p* = .530	*p* = .375	*p* = .202	*p* = .331
*Education*				
No schooling (n = 19)	21.05 (6.77) ^a^	34.53 (7.50)	41.69 (4.44)	5.74 (2.47)
Primary (n = 69)	23.58 (6.71)	36.09 (7.24)	41.26 (5.85) ^a^	6.24 (2.34)
Secondary (n = 51)	24.88 (6.62)	38.68 (6.40)	43.83 (4.65) ^a^	6.27 (2.80)
Technical college (n = 51)	26.19 (5.88) ^a^	39.13 (7.38)	43.68 (4.66)	6.90 (1.89)
One-way ANOVA	*p* = .046*, *ε*^2^ = .05	*p* = .034‡, *ε*^2^ = .05	*p* = .022*, *ε*^2^ = .06	*p* = .444
*Location*				
City (n = 75)	25.12 (6.69)	38.96 (6.78) ^a^	43.93 (3.61) ^a^	6.49 (.28)
Town (n = 55)	23.04 (7.25)	36.40 (6.96)	42.41 (5.31)	6.48 (.32)
Rural area (n = 39)	24.15 (5.41)	35.18 (7.72) ^a^	40.31 (6.80) ^a^	5.67 (.37)
One-way ANOVA	*p* = .212	*p* = .014*, *ε*^2^ = .05	*p* = .039*, *ε*^2^ = .04	*p* = .162
*Employment status*				
Unemployed (n = 58)	22.57 (7.83)	35.51 (7.76)	42.45 (4.74)	5.64 (2.52)
Employed (n = 62)	24.95 (6.25)	38.44 (7.43)	42.43 (6.06)	6.60 (2.49)
Mann-Whitney U test	*p* = .114	*p* = .044*, *d* = .22	*p* = .535	*p* = .038*, *d* = .22

*Note*. ANOVA uses Kruskal-Wallis test;
Dwass-Steel-Critchlow-Flinger pairwise tests were used for post-hoc
comparisons; † Groups were created using a median split and a “no
children” group; ^a^ significant difference between groups when
*p* < .05; *d*
/*ε*^2^ effect size. ‡ Although a
significant difference was detected, there were no significant
differences in the pairwise comparisons.

**Table B7. table7-08862605211028323:** Correlations Between the ARM Factors and Psychosocial Variables in
the Colombian Sample.

	**1. Family Support and Relationships**	**2. Community Support and Belonging**	**3. Contextual Support and Opportunities**	**4. Support From Friends**
1. TEC	-.12	-.08	-.01	-.00
2. CES	.02	.13	.09	.22**
3. Consequences of sexual violence	-.11	-.01	-.05	.14
4. Current problems	-.21**	-.15	-.10	-.13
5. Feeling safe in community	.13	.17*	.06	.08
6. Feeling able to ask for help	.02	.24**	.26**	.13
7. Perceived health	.27**	.22**	.14	.06
8. Perceived QoL	.24**	.25**	.10	.03

*Note*. All correlations are Spearman; **p*
< .05. ***p* < .01. ****p* <
.001.

**Table B8. table8-08862605211028323:** Factor Loadings of the Six-factor Model for Uganda.

	**1. Cultural and Social Bonds**	**2. Familial Bonds**	**3. Individual Strengths**	**4. Cooperation and Community**	**5. Relationships With Friends and Community**	**6. Family Resources and Support**
Item 22	**.67**					
Item 9	**.57**					
Item 23	**.49**				**.35**	
Item 28	**.46**					
Item 10	**.43**					
Item 11	**.42**					
Item 4						
Item 12						
Item 3						
Item 17		**.68**				
Item 24		**.65**				
Item 26		**.41**				
Item 15						
Item 21			**.62**			
Item 25			**.58**			
Item 8			**.49**			
Item 16			**.34**			
Item 13			**.34**			
Item 18						
Item 20						
Item 2				**1.00**		
Item 1				**.40**		
Item 19					**.68**	
Item 27					**.53**	
Item 14					**.32**	
Item 5						**.69**
Item 7						**.63**
Item 6						**.52**

*Note*. Items in bold were retained on the factor.

**Table B9. table9-08862605211028323:** Descriptive Statistics (Mean, SD) for the Factors and Group
Comparisons in the Ugandan Sample.

	**1. Cultural and Social Bonds**	**2. Familial Bonds**	**3. Individual Strengths**	**4. Cooperation and Community**	**5. Relationships With Friends and Community**	**6. Family resources & support**
Overall sample	26.08 (3.59)	12.05 (2.79)	19.25 (3.47)	7.66 (1.84)	14.70 (3.42)	9.70 (2.91)
*Age (median split)*						
<39 (n = 72)	26.20 (4.08)	12.01 (3.07)	19.43 (3.66)	7.64 (1.89)	14.82 (3.47)	9.68 (2.99)
≥39 (n = 78)	25.92 (3.12)	12.05 (2.54)	18.96 (3.26)	7.65 (1.82)	14.50 (3.37)	9.65 (2.82)
Mann-Whitney U test	*p* = .223	*p* = .649	*p* = .325	*p* = .917	*p* = .482	*p* = .839
*Ethnicity*						
Acholi (n = 76)	26.00 (4.17)	11.75 (3.34)	20.07 (3.81)	7.46 (2.22)	13.65 (3.73)	9.36 (3.10)
Lango (n = 76)	26.16 (2.95)	12.36 (2.10)	18.47 (2.94)	7.87 (1.36)	15.72 (2.73)	10.03 (2.67)
Mann-Whitney U test	*p* = .641	*p* = .752	*p* = .003*, *d* = .28	*p* = .656	*p*<.001*, *d* = .36	*p* = .201
*Marital status*						
Not married (n = 34)	26.00 (4.03)	11.44 (3.14)	18.72 (3.63)	7.85 (1.46)	14.42 (3.46)	9.38 (3.03)
Married (n = 62)	25.77 (3.96)	12.95 (2.25)	19.18 (3.39)	7.69 (1.89)	15.26 (3.01)	10.37 (2.72)
Mann-Whitney U test	*p* = .589	*p* = .021*, *d* = .28	*p* = .497	*p* = .925	*p* = .245	*p* = .056
*Number of children* †						
0-3 (n = 53)	27.08 (2.79)	12.08 (2.87)	19.30 (3.53)	7.85 (1.51)	15.44 (3.13)	10.36 (2.97)
4+ (n = 99)	25.55 (3.86)	12.04 (2.76)	19.22 (3.46)	7.57 (2.00)	14.30 (3.51)	9.34 (2.82)
Mann-Whitney U test	*p* = .012*, *d* = .25	.899	.938	.716	*p* = .034*, *d* = .21	.052
*Education*						
No schooling (n = 84)	25.82 (3.93)	12.15 (2.66)	18.94 (3.79)	7.49 (1.89)	14.85 (3.41)	10.00 (2.88)
Primary (n = 63)	26.43 (3.19)	12.00 (3.01)	19.70 (3.04)	7.90 (1.83)	14.48 (3.54)	9.35 (2.95)
Mann-Whitney U test	*p* = .350	*p* = .997	*p* = .261	*p* = .168	*p* = .676	*p* = .177
*Location* ‡						
City/town (n = 34)	25.69 (4.03)	10.85 (3.67)	20.61 (3.62) ^a^	7.29 (2.50)	12.79 (3.81) ^ab^	7.88 (2.86) ^ab^
Trading centre (n = 27)	26.33 (3.60)	11.85 (2.89)	18.70 (3.69)	7.41 (1.60)	15.04 (3.23) ^a^	10.26 (2.98) ^a^
Village (n = 91)	25.69 (4.03)	12.57 (2.21)	18.94 (3.27) ^a^	7.88 (1.60)	15.29 (3.09) ^b^	10.19 (2.65) ^b^
One-way ANOVA	*p* = .939	*p* = .079	*p* = .034*, *ε*^2^ = .05	*p* = .321	*p<*.001*, *ε*^2^ = .09	*p*<.001*, *ε*^2^ = .11
*Employment status*						
Unemployed (n = 84)	26.35 (3.80)	12.42 (2.33)	18.86 (3.73)	7.86 (1.70)	15.05 (3.21)	9.82 (3.04)
Employed (n = 63)	25.84 (3.38)	11.63 (3.35)	19.76 (3.19)	7.35 (2.04)	14.15 (3.73)	9.54 (2.82)
Mann-Whitney U test	*p* = .202	*p* = .348	*p* = .156	*p* = .142	*p* = .150	*p* = .597

*Note*. ANOVA uses Kruskal-Wallis test;
Dwass-Steel-Critchlow-Flinger pairwise tests were used for post-hoc
comparisons; † Groups were created using a median split, though there
were not enough individuals to form a “no children” group; ‡ city and
town groups were combined as there were too few individually;
^ab^ significant difference between groups when
*p* < .05; *ε*^2^ effect
size.

**Table B10. table10-08862605211028323:** Correlations Between the ARM Factors and Psychosocial Variables in
the Ugandan Sample.

	**1. Cultural and Social Bonds**	**2. Familial Bonds**	**3. Individual Strengths**	**4. Cooperation and Community**	**5. Relationships With Friends and Community**	**6. Family Resources and Support**
1. TEC	.16	-.09	.00	-.01	.14	-.06
2. CES	.22**	.06	-.12	.08	.23**	-.02
3. Consequences of sexual violence	.05	-.25**	-.20*	-.03	-.05	-.09
4. Current problems	.05	-.18*	-.20*	-.04	.03	-.09
5. Feeling safe in community	.11	.13	.22**	.22**	.08	.13
6. Feeling able to ask for help	.00	.26**	.01	.07	.26**	.19*
7. Perceived health	.02	.05	.17*	-.04	-.04	.02
8. Perceived QoL	.06	.18*	.23**	.04	.02	.16

*Note*. All correlations are Spearman; **p*
< .05. ***p* < .01. ****p* <
.001.

## Appendix C: Scales

1.
**Adult Resilience Measure (Resilience Research Centre, 2006)**


**Table table11-08862605211028323:** 

**To what extent do each of the statements below describe you?**	*Not at all*	*A little*	*Some* what	*Quite* a bit	*A* lot
1. I have people I can respect in my life					
2. I cooperate with people around me					
3. Getting and improving qualifications or skills is important to me					
4. I know how to behave in different social situations					
5. My family have usually supported me through life					
6. My family know a lot about me					
7. If I am hungry, I can get food to eat					
8. I try to finish what I start					
9. Spiritual beliefs are a source of strength for me					
10. I am proud of my ethnic background					
11. People think that I am fun to be with					
12. I talk to my family/partner about how I feel					
13. I can solve problems without harming myself or others					
14. I feel supported by my friends					
15. I know where to get help in my community					
16. I feel I belong in my community					
17. My family stands by me during difficult times					
18. My friends stand by me during difficult times					
19. I am treated fairly in my community					
20. I have opportunities to show others that I can act responsibly					
21. I am aware of my own strengths					
22. I participate in organized religious activities					
23. I think it is important to support my community					
24. I feel secure when I am with my family					
25. I have opportunities to apply my abilities in life (life skills, a job, caring for others)					
26. I enjoy my family’s/partner’s cultural and family traditions					
27. I enjoy my community’s culture and traditions					
28. I am proud to be a citizen of…					

2.
**Traumatic Events Checklist**


**Table table12-08862605211028323:** 

**Which of the following situations have you experienced during war/armed conflict in your country?**	*No*	*Yes*	*Prefer not to say*
1. Been forcibly displaced from your home/community			
2. Witnessed (i.e. seen) your home being destroyed			
3. Lived in temporary accommodation for displaced persons			
4. Been unable to feed yourself or your family			
5. Been forcibly separated from your family			
6. Been seriously injured/wounded			
7. Been abducted/kidnapped			
8. Been forcibly detained in a camp			
9. Experienced the death of a child			
10. Had members of your family ‘disappear’ (go missing)			
11. Had members of your family killed			
12. Witnessed (i.e. seen) people being beaten or tortured			
13. Witnessed (i.e. seen) people being killed			
14. Experienced torture (physical or psychological)			
15. Experienced sexual violence (including rape, forced marriage, forced pregnancy, sexual enslavement, forced abortion, sexual torture or genital beatings)			
16. Witnessed (i.e. seen) an act of rape or sexual violence			
17. Been forcibly recruited into an armed group			
18. Been forced to participate in a massacre, act of torture, abduction, rape, etc.			
19. Been forced to participate in acts of looting/plunder			
20. Been betrayed by a family member or neighbor during the war			
21. If you answered YES to more than one of the items above, which is the most distressing to you now?			
22. How long ago did the most distressing event happen?			

3.
**Centrality of Event Scale (short version; Berntsen & Rubin, 2006)**


**Table table13-08862605211028323:** 

**Thinking specifically about the sexual violence that you experienced during the war/armed conflict in your country, to what extent do you disagree or agree with the following statements?**	*Totally disagree*	Disagree	*Neither agree nor disagree*	Agree	Totally agree
1. I feel that this event (i.e. sexual violence) has become part of my identity [Explanation: The sexual violence has become part of how I define myself as a person]					
2. This event has become a reference point for the way I understand myself and the world *To explain myself and the world around me, I always refer back to the sexual violence I experienced]*					
3. I feel that this event has become a central part of my life story *If I were to tell the story of my life, my experience of sexual violence would be a central event]*					
4. This event has colored the way I think and feel about other experiences [*Explanation:**My experience of sexual violence has affected how I think and feel about other things that happen in my life]*					
5. This event permanently changed my life *The sexual violence has had a lasting impact on my life]*					
6. I often think about the effects this event will have on my future					
7. This event was a turning point in my life *The sexual violence took my life in a new direction]*					

4.
**Consequences of Sexual Violence Scale**


**Table table14-08862605211028323:** 

**What have been the main consequences of the sexual violence that you experienced during the war/armed conflict in your country?**	*No*	*Yes*
1. Problems with body image		
2. Low self-esteem		
3. Altered sexual desire (e.g. loss of sexual desire, increased sexual desire, etc.)		
4. Difficulty trusting other people		
5. Sense of guilt/self-blame		
6. Child/children born of rape		
7. HIV/AIDS		
8. Other sexually transmitted infections (e.g. syphilis)		
9. Gynecological problems		
10. Stigmatization (e.g. insults/abuse from the community, social exclusion, etc.)		
11. Rejection by family		
12. Broken relationships		
13. Other		

4.
**Current Life Problems**


**Table table15-08862605211028323:** 

**What are the principal problems that you face today?**	*No*	*Yes*
1. Physical health problems (e.g. high blood pressure, diabetes, chronic pain, heart conditions, cancer, etc.)		
2. Psychological problems (e.g. depression, anxiety, nightmares, insomnia, mood swings, etc.)		
3. Economic insecurity/poverty		
4. Unemployment		
5. Housing problems (e.g. unable to pay rent, poor living conditions, don’t have own home)		
6. Land issues (e.g. lack of access to land, unable to return to own land, etc.)		
7. Living as an internally displaced person		
8. Difficulty in meeting basic everyday needs (e.g. water, food, electricity, sanitation, clothing)		
9. Lack of access to healthcare		
10. Lack of access to education (for self or children)		
11. Problems with partner		
12. Other family and relationship problems		
13. Abuse/bullying from community members		
14. Loneliness		
15. Addictions (e.g. alcoholism)		
16. Domestic violence		
17. Threats (e.g. death threats, threats against family members)		
18. Other (please specify)		

5.
**Life Today**


**Table table16-08862605211028323:** 

**Do you feel safe in your community?**				
1. Never	2. Occasionally	3. Sometimes	4. Most of the time	5. Always
**Do you feel able to ask for help when you need it?**				
1. Never	2. Occasionally	3. Sometimes	4. Most of the time	5. Always
**In general, how would you rate your health?**				
1. Poor	2. Fair	3. Good	4. Very good	5. Excellent
**How would you rate your quality of life?**				
1. Poor	2. Fair	3. Good	4. Very good	5. Excellent

## References

BerntsenD., & RubinD.C. (2006). The
centrality of event scale: A measure of integrating a trauma into one’s
identity and its relation to post-traumatic stress disorder
symptoms. *Behaviour Research &
Therapy*, 44(2),
219-231.
https://doi.org/10.1016/j.brat.2005.01.0091638906210.1016/j.brat.2005.01.009PMC3974102Resilience Research
Centre. (2016). *The Resilience
Research Centre Adult Resilience Measure (RRC-ARM): User’s manual –
Research, May 2016*.
https://cyrm.resilienceresearch.org/files/ArchivedMaterials.zip

## Supplemental Material

Supplemental material for this article is available online.Click here for additional data file.Supplemental Material for Measuring Resilience in the Context of Conflict-Related
Sexual Violence: A Novel Application of the Adult Resilience Measure (ARM) by
Janine Natalya Clark, Philip Jefferies, Sarah Foley, and Michael Ungar, in
Journal of Interpersonal Violence
