# Supplementary material for: Measuring Resilience in the Context of Conflict-Related Sexual Violence: A Novel Application of the Adult Resilience Measure (ARM)
Source: J Interpers Violence. 2021 Jul 7;37(19-20):NP17570–615. doi: 10.1177/08862605211028323 (PMC9554281; doi:10.1177/08862605211028323)
Supplement: Supplemental material for this article is available online. [file sj-pdf-1-jiv-10.1177_08862605211028323.pdf]

Appendix A: Confirmatory and Exploratory Factor Analyses

Confirmatory factor analysis (CFA)

Given that other studies employing the ARM have found varying factor structures (e.g., Arslan, 2015; Liebenberg & Moore, 2018), our first aim was to use CFA to assess the conceptual and measurement equivalence of the ARM factor structure across sites. To evaluate the CFA, we used a maximum likelihood estimator and evaluated model fit using the established criteria of a Comparative Fit Index (CFI) and Tucker-Lewis Index (TLI) >.90 (Hu & Bentler, 1999) and a Root Mean Square Error of Approximation (RMSEA) and Standardised Root Mean Square Residual (SRMR) <.08 (Hu & Bentler, 1999).

An initial CFA applied to the entire dataset resulted in poor fit (CFI=.69, TLI=.66, RMSEA=.08 [90% =.07-.08], SRMR=.08). When checking the fit per country, similar poor fit statistics were observed: BiH: CFI=.70, TLI=.67, RMSEA=.09, [90% CI=.08-.10], SRMR=.10; Colombia: CFI=.69, TLI=.66, RMSEA=.09, [90% CI=.08-.09], SRMR=.08; Uganda: CFI=.57, TLI=.53, RMSEA=.09, [90% CI=.08-.10], SRMR=.09). Although reviewing the modification indices suggested some ways in which the model could be improved (by freeing parameters), these improvements still did not result in a model with adequate fit, suggesting the original three-factor structure of the ARM should be reconsidered.

Exploratory Factor Analysis (EFA)

We accordingly revisited the factor structure of the ARM through EFA to determine a better-fitting model. We chose to use EFA (rather than principle components analysis) to identify

the underlying dimensions of the measure (for other examples of EFAs applied to the CYRM/ARM, see Robinson et al., 2016; Amini-Tehrani et al., 2020; Kaunda-Khangamwa et al., 2020). While similar to PCA, EFA is widely considered as the appropriate approach when investigating the dimensionality of social and psychological constructs because, unlike PCA, it takes account of measurement error and shared variance (Brown, 2006).

Given the variation in the CFA fit statistics for each country sample, and the variation in factor structures when the ARM has been used in other countries (e.g., see van Rensburg et al. 2017; Liebenberg & Moore, 2018), we determined that individual EFAs for each country would result in the most contextually appropriate solutions. For each country sample, Barlett's Test of Sphericity produced a significant finding (p <.001), indicating interrelationships between the variables (Field, 2009), and a Kaiser-Meyer-Olkin test for sampling adequacy confirmed that values fell between .6 and 1.0 (Tabachnick & Fidell, 2006) (BiH = .77; Colombia = .77; Uganda = .73).

For the EFAs, we used a maximum likelihood extraction technique and an oblique rotation strategy (oblimin), given that others have found highly correlated factors in previous structural investigations of the CYRM and ARM (e.g., Liebenberg et al., 2012). To determine factor structure we used Comrey and Lee's generally accepted thresholds for item loading values, where items loading ≥.32 are considered the minimum values for loading. Items that cross load (loadings ≥.32 on two or more factors) can be managed in various ways (see Yong & Pearce, 2013). Some suggest that a minimum separation between factor loadings indicates how to manage an item (Howard, 2016; Matsunaga, 2010), while others retain cross-loading items regardless (e.g., Le & Cheong, 2010). We reviewed each cross-loading item to see if the loading separation suggested that an item could be dropped from a particular factor. However, we were also open to retaining cross-loading items, given that some of the items in the ARM were likely to relate to multiple dimensions of resilience.

We then used multiple criteria to assess and select an appropriate model; including examining scree plots and eigenvalues, RMSEA values <.08 (Hu and Bentler, 1999), ensuring factors correlated appropriately and also Henson and Robert's (2006) 'reasoned reflection' (p. 399) concerning sensible configurations of the items per factor in factor loading matrices. In sum, we sought a parsimonious model for each country that had good statistical properties and one that possessed relatively clear and distinct factors.

References

Amini-Tehrani, M., Nasiri, M., Sadeghi, R., Hoseini, E.-S., Jalali, T., & Zamanian, H. (2020). Social-ecological measure of resilience: An adapted measure for Persian-speaking university students. *Health Promotion Perspectives, 10*(3), 207–219. https://doi.org/10.34172/hpp.2020.xx

Arslan, G. (2015). Psychometric properties of Adult Resilience Measure (ARM): The study of reliability and validity. *Ege Eğitim Dergisi*, *16*(2), 344–357.

Brown, T. A. (2006). *Confirmatory factor analysis for applied research*. Guilford Press.

Field, A. (2009). *Discovering statistics using SPSS: Third edition* (3rd ed.). Sage Publications.

Henson, R. K., & Roberts, J. K. (2006). Use of exploratory factor analysis in published research: Common errors and some comment on improved practice. *Educational and Psychological Measurement*, *66*(3), 393–416. https://doi.org/10.1177/0013164405282485

Howard, M. C. (2016). A review of exploratory factor analysis decisions and overview of current practices: What we are doing and how can we improve? *International Journal of Human–Computer Interaction*, *32*(1), 51–62. https://doi.org/10.1080/10447318.2015.1087664

Hu, L., & Bentler, P. M. (1999). Cutoff criteria for fit indexes in covariance structure analysis: Conventional criteria versus new alternatives. *Structural Equation Modeling: A Multidisciplinary Journal*, *6*(1), 1–55. https://doi.org/10.1080/10705519909540118

Kaunda-Khangamwa, B. N., Maposa, I., Dambe, R., Malisita, K., Mtagalume, E., Chigaru, L., Munthali, A., Chipeta, E., Phiri, S., & Manderson, L. (2020). Validating a Child Youth Resilience Measurement (CYRM-28) for adolescents living with HIV (ALHIV) in urban Malawi. *Frontiers in Psychology*, *11*. https://doi.org/10.3389/fpsyg.2020.01896

Le, T. C., & Cheong, F. (2010). Perceptions of risk and risk management in Vietnamese catfish farming: An empirical study. *Aquaculture Economics & Management*, *14*(4), 282–314. https://doi.org/10.1080/13657305.2010.526019

Liebenberg, L., & Moore, J.C. (2018). A social ecological measure of resilience for adults: The RRC-ARM. *Social Indicators Research*, *136*, 1–19. https://doi.org/10.1007/s11205-016-1523-y

Liebenberg, L., Ungar, M., & van de Vijver, F. (2012). Validation of the Child and Youth Resilience Measure-28 (CYRM-28) among Canadian youth. *Research on Social Work Practice*, *22*(2), 219–226. https://doi.org/10.1177/1049731511428619

Matsunaga, M. (2010). How to factor-analyze your data right: Do's, don'ts, and how-to's. *International Journal of Psychological Research*, *3*(1), 97–110. https://doi.org/10.21500/20112084.854

Robinson, R. V., David, E. J. R., & Hill, M. (2016). Participatory mixed methods research across cultures. In L.A. Jason & D.S. Glenwick (Eds.), *Handbook of methodological approaches to community-based research: Qualitative, quantitative, and mixed methods* (pp. 273–282). Oxford University Press.

Tabachnick, B. G., & Fidell, L. S. (2006). *Using multivariate statistics* (5th ed.). Allyn & Bacon.

van Rensburg, A. C., Theron, L. C., & Ungar, M. (2017). Using the CYRM-28 with South African young people: A factor structure analysis. *Research on Social Work Practice*, 1–10. https://doi.org/10.1177/1049731517710326

Yong, A. G., & Pearce, S. (2013). A beginner's guide to factor analysis: Focusing on exploratory factor analysis. *Tutorials in Quantitative Methods for Psychology*, *9*(2), 79–94.

Appendix B: Tables

Table 1: Respondents (n = 449) by ethnicity

**BiH**	**Colombia**	**Uganda**
Bosniak *n* = 85	Afro-Colombian *n* = 49	Acholi *n* = 76
Serb *n* = 30	Mestizo *n* = 44	Lango *n* = 76
Croat *n* = 6	Indigenous *n* = 19	
Other *n* = 5	Other *n* = 47	
	Did not understand *n* = 12	

Table 2. Factor loadings of the four-factor model for BiH

	**1. Social and community relations**	**2. Family support & relationships**	**3. Cultural participation & belonging**	**4. Abilities and opportunities**
Item 18	**.73**			
Item 19	**.71**			
Item 16	**.61**		.33	
Item 15	**.60**			
Item 14	**.55**			
Item 21	**.50**			
Item 23	**.41**			
Item 11	**.34**			
Item 2				
Item 17		**.91**		
Item 5		**.88**		
Item 24		**.76**		
Item 6		**.34**		
Item 12		**.32**		

Item 3				
Item 26			**.80**	
Item 27	.36		**.72**	
Item 28			**.46**	
Item 22			**.45**	
Item 25			**.44**	**.34**
Item 10			**.40**	
Item 9				
Item 4				**.60**
Item 13				**.56**
Item 8				**.41**
Item 7				
Item 20				
Item 1				

Note. Items in bold were retained on the factor.

Table 3. Descriptive statistics (Mean, SD) for the factors and group comparisons in the BiH sample

	**1. Social and community relations**	**2. Family support & relationships**	**3. Cultural participation & belonging**	**4. Abilities and opportunities**
Overall sample	30.34 (5.95)	20.44 (4.14)	24.36 (4.21)	16.83 (2.59)
Age (median split)				
<55 (n=58)	29.58 (6.43)	20.26 (4.40)	24.23 (4.15)	17.05 (2.50)
≥55 (n=68)	30.99 (5.49)	20.60 (3.93)	24.47 (4.29)	16.63 (2.66)
Mann-Whitney U test	*p*=.315	*p*=.935	*p*=.661	*p*=.376
Ethnicity ‖				
Bosniak (n=84)	29.93 (6.28)	20.13 (4.42)	24.58 (6.06)	16.58 (2.39)
Serbian (n=30)	31.67 (4.97)	21.50 (3.17)	24.33 (3.34)	17.33 (2.02)
Mann-Whitney U test	*p*=.302	*p*=.208	*p*=.491	*p*=.295
Marital status				
Not married (n=24)	29.96 (4.95)	20.00 (4.29)	23.29 (5.55)	16.33 (2.84)
Married (n=65)	30.37 (5.72)	20.42 (3.96)	24.16 (3.94)	16.98 (2.42)
Mann-Whitney U test	*p*=.694	*p*=.714	*p*=.796	*p*=.375
Number of children †				

None (n=25)	32.00 (4.90)	19.56 (5.29)	23.32 (5.44)	16.04 (2.73)
1 (n=20)	29.80 (6.67)	21.00 (3.87)	24.80 (3.62)	17.15 (2.28)
2+ (n=81)	29.99 (6.04)	20.58 (3.80)	24.57 (3.90)	16.99 (2.60)
One-way ANOVA	*p*=.299	*p*=.692	*p*=.687	*p*=.235
Education ‡				
High school (n=58)	30.48 (9.76)	19.83 (4.40)	24.74 (4.29)	16.86 (2.66)
University (n=51)	29.96 (5.13)	21.20 (3.46)	23.41 (4.28)	16.65 (2.53)
Mann-Whitney U test	*p*=.462	*p*=.127	*p*=.072	*p*=.559
Location §				
Town (n=44)	30.43 (5.41)	19.66 (4.70)	23.20 (4.35)	16.59 (.264)
Suburbs (n=44)	30.00 (5.79)	20.50 (3.45)	24.74 (4.57)	16.80 (2.81)
Village (n=33)	30.06 (6.83)	21.03 (4.33)	25.15 (3.55)	17.06 (2.33)
One-way ANOVA	*p*=.967	*p*=.272	*p*=.050	*p*=.760
Employment status				
Unemployed (n=91)	30.23 (6.07)	20.31 (4.07)	24.43 (4.35)	17.00 (2.78)
Employed (n=25)	30.76 (5.00)	21.28 (4.27)	24.20 (4.02)	18.00 (1.87)
Mann-Whitney U test	*p*=.833	*p*=.146	*p*=.703	*p*=.233

Note: ANOVA uses Kruskal-Wallis test; † Groups were created using a median split and a ‘no children’ group; ‡ No participants reported completing only primary school or not completing primary school; § Only five participants reported living in a city, so were excluded from the comparative analysis. ‖ Six individuals identified as Croat and five as ‘other’, but these groups were small and so excluded from the comparative analysis.

Table 4. Correlations between the ARM factors and psychosocial variables in the BiH sample

	**1. Social and community relations**	**2. Family support & relationships**	**3. Cultural participation & belonging**	**4. Abilities and opportunities**
1. TEC	.08	.02	.24**	.10
2. CES	.06	.00	.17	.05
3. Consequences of sexual violence	-.14	-.22*	-.21*	-.10
4. Current problems	-.17	-.23**	-.22*	-.08
5. Feeling safe in community	.31***	.32***	.32***	.30***
6. Feeling able to ask for help	.40***	.39***	.31***	.25**
7. Perceived health	.11	.18*	-.01	.04
8. Perceived QoL	.18*	.28**	.07	.07

Note: All correlations are Spearman; *p<.05, **p<.01, ***p<.001.

Table 5. Factor loadings of the four-factor model for Colombia

	1. Family support & relationships	2. Community support & belonging	3. Contextual support & opportunities	4. Support from friends
Item 17	**.81**			
Item 5	**.79**			
Item 6	**.73**			
Item 24	**.65**			
Item 12	**.47**	.34		
Item 7	**.35**			
Item 27		**.61**		
Item 25		**.55**		
Item 15		**.49**		
Item 16		**.46**		
Item 26	.35	**.44**		
Item 23		**.40**		
Item 20		**.38**		
Item 19		**.36**		
Item 21		**.35**	.33	
Item 22				
Item 28				
Item 4			**.61**	
Item 1			**.57**	
Item 3			**.50**	
Item 11			**.45**	
Item 9			**.45**	
Item 2			**.40**	
Item 10			**.39**	
Item 8			**.35**	
Item 13			**.32**	
Item 14				**1.01**
Item 18				**.71**

Note. Items in bold were retained on the factor.

Table 6. Descriptive statistics (Mean, SD) for the factors and group comparisons in the Colombian sample

	**1. Family support & relationships**	**2. Community support & belonging**	**3. Contextual support & opportunities**	**4. Support from friends**
Overall sample	24.16 (6.65)	37.24 (7.18)	42.53 (5.26)	6.32 (2.43)
Age (median split)				
<42 (n=79)	28.34 (5.45)	17.13 (5.39)	23.00 (4.04)	16.10 (2.73)
≥42 (n=91)	30.02 (6.59)	16.76 (5.43)	24.08 (3.86)	16.52 (2.70)
Mann-Whitney U test	p=.057	p=.679	p=.137	p=.289
Ethnicity				
Afro-Colombian (n=49)	24.61 (6.22)	37.24 (7.31)	42.90 (5.04)	5.90 (2.50)
Indigenous (n=19)	23.74 (6.33)	35.89 (6.21)	41.50 (6.56)	6.63 (1.71)
Mestizo (n=44)	24.98 (6.70)	37.98 (8.43)	41.61 (6.21)	6.50 (2.57)
'Other' (n=47)	23.62 (7.04)	37.36 (6.22)	43.15 (3.83)	6.45 (2.49)
One-way ANOVA	p=.769	p=.748	p=.477	p=.517
Marital status				
Not married (n=65)	23.17 (7.00)	35.98 (6.94)	42.52 (4.87)	6.11 (2.59)
Married (n=21)	23.75 (6.48)	35.26 (9.66)	42.21 (5.18)	6.29 (2.37)
Mann-Whitney U test	p=.733	p=.764	p=.817	p=.772
Number of children †				
None (n=13)	22.38 (7.07)	34.92 (7.58)	41.31 (3.88)	5.23 (2.31)
1-2 (n=49)	23.53 (7.88)	36.94 (7.58)	42.32 (5.02)	6.24 (2.45)
3+ (n=108)	24.65 (5.99)	37.59 (7.02)	42.74 (5.54)	6.44 (2.31)
One-way ANOVA	p=.530	p=.375	p=.202	p=.331
Education				
No schooling (n=19)	21.05 (6.77) [a]	34.53 (7.50)	41.69 (4.44)	5.74 (2.47)
Primary (n=69)	23.58 (6.71)	36.09 (7.24)	41.26 (5.85) [a]	6.24 (2.34)
Secondary (n=51)	24.88 (6.62)	38.68 (6.40)	43.83 (4.65) [a]	6.27 (2.80)
Technical college (n=51)	26.19 (5.88) [a]	39.13 (7.38)	43.68 (4.66)	6.90 (1.89)
One-way ANOVA	p=.046*, ε^2=.05	p=.034‡, ε^2=.05	p=.022*, ε^2=.06	p=.444
Location				
City (n=75)	25.12 (6.69)	38.96 (6.78) [a]	43.93 (3.61) [a]	6.49 (.28)
Town (n=55)	23.04 (7.25)	36.40 (6.96)	42.41 (5.31)	6.48 (.32)
Rural area (n=39)	24.15 (5.41)	35.18 (7.72) [a]	40.31 (6.80) [a]	5.67 (.37)
One-way ANOVA	p=.212	p=.014*, ε^2=.05	p=.039*, ε^2=.04	p=.162

Employment status				
Unemployed (n=58)	22.57 (7.83)	35.51 (7.76)	42.45 (4.74)	5.64 (2.52)
Employed (n=62)	24.95 (6.25)	38.44 (7.43)	42.43 (6.06)	6.60 (2.49)
Mann-Whitney U test	*p*=.114	*p*=.044*, *d*=.22	*p*=.535	*p*=.038*, *d*=.22

Note: ANOVA uses Kruskal-Wallis test; Dwass-Steel-Critchlow-Flinger pairwise tests were used for post-hoc comparisons; † Groups were created using a median split and a 'no children' group; [a] significant difference between groups when $p<.05$; *d* /ε^2 effect size. ‡ Although a significant difference was detected, there were no significant differences in the pairwise comparisons.

Table 7. Correlations between the ARM factors and psychosocial variables in the Colombian sample

	**1. Family support & relationships**	**2. Community support & belonging**	**3. Contextual support & opportunities**	**4. Support from friends**
1. TEC	-.12	-.08	-.01	-.00
2. CES	.02	.13	.09	.22**
3. Consequences of sexual violence	-.11	-.01	-.05	.14
4. Current problems	-.21**	-.15	-.10	-.13
5. Feeling safe in community	.13	.17*	.06	.08
6. Feeling able to ask for help	.02	.24**	.26**	.13
7. Perceived health	.27**	.22**	.14	.06
8. Perceived QoL	.24**	.25**	.10	.03

Note: All correlations are Spearman; *$p<.05$, **$p<.01$, ***$p<.001$.

Table 8. Factor loadings of the six-factor model for Uganda

	**1. Cultural & social bonds**	**2. Familial bonds**	**3. Individual strengths**	**4. Cooperation & community**	**5. Relationships with friends & community**	**6. Family resources & support**
Item 22	**.67**					
Item 9	**.57**					
Item 23	**.49**				.35	
Item 28	**.46**					
Item 10	**.43**					
Item 11	**.42**					

Item 4					
Item 12					
Item 3					
Item 17	**.68**				
Item 24	**.65**				
Item 26	**.41**				
Item 15					
Item 21		**.62**			
Item 25		**.58**			
Item 8		**.49**			
Item 16		**.34**			
Item 13		**.34**			
Item 18					
Item 20					
Item 2			**1.00**		
Item 1			**.40**		
Item 19				**.68**	
Item 27				**.53**	
Item 14				**.32**	
Item 5					**.69**
Item 7					**.63**
Item 6					**.52**

Note. Items in bold were retained on the factor.

Table 9. *Descriptive statistics (Mean, SD) for the factors and group comparisons in the Ugandan sample*

	**1. Cultural & social bonds**	**2. Familial bonds**	**3. Individual strengths & community support**	**4. Cooperation & community**	**5. Relationships with friends & community**	**6. Family resources & support**
Overall sample	26.08 (3.59)	12.05 (2.79)	19.25 (3.47)	7.66 (1.84)	14.70 (3.42)	9.70 (2.91)
Age (median split)						
<39 (n=72)	26.20 (4.08)	12.01 (3.07)	19.43 (3.66)	7.64 (1.89)	14.82 (3.47)	9.68 (2.99)
≥39 (n=78)	25.92 (3.12)	12.05 (2.54)	18.96 (3.26)	7.65 (1.82)	14.50 (3.37)	9.65 (2.82)
Mann-Whitney U test	*p*=.223	*p*=.649	*p*=.325	*p*=.917	*p*=.482	*p*=.839
Ethnicity						
Acholi (n=76)	26.00 (4.17)	11.75 (3.34)	20.07 (3.81)	7.46 (2.22)	13.65 (3.73)	9.36 (3.10)
Lango (n=76)	26.16 (2.95)	12.36 (2.10)	18.47 (2.94)	7.87 (1.36)	15.72 (2.73)	10.03 (2.67)
Mann-Whitney U test	*p*=.641	*p*=.752	*p*=.003*, *d*=.28	*p*=.656	*p*<.001*, *d*=.36	*p*=.201
Marital status						
Not married (n=34)	26.00 (4.03)	11.44 (3.14)	18.72 (3.63)	7.85 (1.46)	14.42 (3.46)	9.38 (3.03)
Married (n=62)	25.77 (3.96)	12.95 (2.25)	19.18 (3.39)	7.69 (1.89)	15.26 (3.01)	10.37 (2.72)
Mann-Whitney U test	*p*=.589	*p*=.021*, *d*=.28	*p*=.497	*p*=.925	*p*=.245	*p*=.056
Number of children †						
0-3 (n=53)	27.08 (2.79)	12.08 (2.87)	19.30 (3.53)	7.85 (1.51)	15.44 (3.13)	10.36 (2.97)
4+ (n=99)	25.55 (3.86)	12.04 (2.76)	19.22 (3.46)	7.57 (2.00)	14.30 (3.51)	9.34 (2.82)
Mann-Whitney U test	*p*=.012*, *d*=.25	.899	.938	.716	*p*=.034*, *d*=.21	.052

Education						
No schooling (n=84)	25.82 (3.93)	12.15 (2.66)	18.94 (3.79)	7.49 (1.89)	14.85 (3.41)	10.00 (2.88)
Primary (n=63)	26.43 (3.19)	12.00 (3.01)	19.70 (3.04)	7.90 (1.83)	14.48 (3.54)	9.35 (2.95)
Mann-Whitney U test	p=.350	p=.997	p=.261	p=.168	p=.676	p=.177
Location ‡						
City/town (n=34)	25.69 (4.03)	10.85 (3.67)	20.61 (3.62) [a]	7.29 (2.50)	12.79 (3.81) [ab]	7.88 (2.86) [ab]
Trading centre (n=27)	26.33 (3.60)	11.85 (2.89)	18.70 (3.69)	7.41 (1.60)	15.04 (3.23) [a]	10.26 (2.98) [a]
Village (n=91)	25.69 (4.03)	12.57 (2.21)	18.94 (3.27) [a]	7.88 (1.60)	15.29 (3.09) [b]	10.19 (2.65) [b]
One-way ANOVA	p=.939	p=.079	p=.034*, ε^2=.05	p=.321	p<.001*, ε^2=.09	p<.001*, ε^2=.11
Employment status						
Unemployed (n=84)	26.35 (3.80)	12.42 (2.33)	18.86 (3.73)	7.86 (1.70)	15.05 (3.21)	9.82 (3.04)
Employed (n=63)	25.84 (3.38)	11.63 (3.35)	19.76 (3.19)	7.35 (2.04)	14.15 (3.73)	9.54 (2.82)
Mann-Whitney U test	p=.202	p=.348	p=.156	p=.142	p=.150	p=.597

Note: ANOVA uses Kruskal-Wallis test; Dwass-Steel-Critchlow-Flinger pairwise tests were used for post-hoc comparisons; † Groups were created using a median split, though there were not enough individuals to form a 'no children' group; ‡ city and town groups were combined as there were too few individually; [ab] significant difference between groups when p<.05; ε^2 effect size.

Table 10. *Correlations between the ARM factors and psychosocial variables in the Ugandan sample*

	**1. Cultural & social bonds**	**2. Familial bonds**	**3. Individual strengths & community support**	**4. Cooperation & community**	**5. Relationships with friends & community**	**6. Family resources & support**
1. TEC	.16	-.09	.00	-.01	.14	-.06
2. CES	.22**	.06	-.12	.08	.23**	-.02
3. Consequences of sexual violence	.05	-.25**	-.20*	-.03	-.05	-.09
4. Current problems	.05	-.18*	-.20*	-.04	.03	-.09
5. Feeling safe in community	.11	.13	.22**	.22**	.08	.13
6. Feeling able to ask for help	.00	.26**	.01	.07	.26**	.19*
7. Perceived health	.02	.05	.17*	-.04	-.04	.02
8. Perceived QoL	.06	.18*	.23**	.04	.02	.16

Note: All correlations are Spearman; *p<.05, **p<.01, ***p<.001.

Appendix C: Scales

1. Adult Resilience Measure (Resilience Research Centre, 2006)

**To what extent do each of the statements below describe you?**	*Not at all*	*A little*	*Some what*	*Quite a bit*	*A lot*
1. I have people I can respect in my life					
2. I cooperate with people around me					
3. Getting and improving qualifications or skills is important to me					
4. I know how to behave in different social situations					
5. My family have usually supported me through life					
6. My family know a lot about me					
7. If I am hungry, I can get food to eat					
8. I try to finish what I start					
9. Spiritual beliefs are a source of strength for me					
10. I am proud of my ethnic background					
11. People think that I am fun to be with					
12. I talk to my family/partner about how I feel					
13. I can solve problems without harming myself or others					
14. I feel supported by my friends					
15. I know where to get help in my community					
16. I feel I belong in my community					
17. My family stands by me during difficult times					
18. My friends stand by me during difficult times					
19. I am treated fairly in my community					
20. I have opportunities to show others that I can act responsibly					

21. I am aware of my own strengths					
22. I participate in organized religious activities					
23. I think it is important to support my community					
24. I feel secure when I am with my family					
25. I have opportunities to apply my abilities in life (life skills, a job, caring for others)					
26. I enjoy my family's/partner's cultural and family traditions					
27. I enjoy my community's culture and traditions					
28. I am proud to be a citizen of…					

2. Traumatic Events Checklist

**Which of the following situations have you experienced during war/armed conflict in your country?**	*No*	*Yes*	*Prefer not to say*
1. Been forcibly displaced from your home/community			
2. Witnessed (i.e. seen) your home being destroyed			
3. Lived in temporary accommodation for displaced persons			
4. Been unable to feed yourself or your family			
5. Been forcibly separated from your family			
6. Been seriously injured/wounded			
7. Been abducted/kidnapped			
8. Been forcibly detained in a camp			
9. Experienced the death of a child			
10. Had members of your family 'disappear' (go missing)			
11. Had members of your family killed			
12. Witnessed (i.e. seen) people being beaten or tortured			
13. Witnessed (i.e. seen) people being killed			

14. Experienced torture (physical or psychological)			
15. Experienced sexual violence (including rape, forced marriage, forced pregnancy, sexual enslavement, forced abortion, sexual torture or genital beatings)			
16. Witnessed (i.e. seen) an act of rape or sexual violence			
17. Been forcibly recruited into an armed group			
18. Been forced to participate in a massacre, act of torture, abduction, rape, etc.			
19. Been forced to participate in acts of looting/plunder			
20. Been betrayed by a family member or neighbour during the war			
21. If you answered YES to more than one of the items above, which is the one most distressing to you now?			
22. How long ago did the most distressing event happen?			

3. Centrality of Event Scale (short version) (Berntsen & Rubin, 2006)

**Thinking specifically about the sexual violence that you experienced during the war/armed conflict in your country, to what extent do you disagree or agree with the following statements?**	*Totally disagree*	*Disagree*	*Neither agree nor disagree*	*Agree*	*Totally agree*
1. I feel that this event (i.e. sexual violence) has become part of my identity<br><br>[Explanation: *The sexual violence has become part of how I define myself as a person]*					
2. This event has become a reference point for the way I understand myself and the world<br><br>[Explanation: *To explain myself and the world around me, I always refer back to the sexual violence I experienced]*					

3. I feel that this event has become a central part of my life story<br><br>*[<u>Explanation:</u> If I were to tell the story of my life, my experience of sexual violence would be a central event]*					
4. This event has coloured the way I think and feel about other experiences<br><br>[*<u>Explanation:</u> My experience of sexual violence has affected how I think and feel about other things that happen in my life]*					
5. This event permanently changed my life<br><br>*[<u>Explanation:</u> The sexual violence has had a lasting impact on my life]*					
6. I often think about the effects this event will have on my future					
7. This event was a turning point in my life<br><br>*[<u>Explanation:</u> The sexual violence took my life in a new direction]*					

4. Consequences of Sexual Violence Scale

**What have been the main consequences of the sexual violence that you experienced during the war/armed conflict in your country?**	*No*	*Yes*
1. Problems with body image		
2. Low self-esteem		
3. Altered sexual desire (e.g. loss of sexual desire, increased sexual desire, etc.)		
4. Difficulty trusting other people		
5. Sense of guilt/self-blame		
6. Child/children born of rape		
7. HIV/AIDS		
8. Other sexually transmitted infections (e.g. syphilis)		
9. Gynaecological problems		
10. Stigmatization (e.g. insults/abuse from the community, social exclusion, etc.)		
11. Rejection by family		

12. Broken relationships		
13. Other		

4. Current Life Problems

**What are the principal problems that you face today?**	*No*	*Yes*
1. Physical health problems (e.g. high blood pressure, diabetes, chronic pain, heart conditions, cancer, etc.)		
2. Psychological problems (e.g. depression, anxiety, nightmares, insomnia, mood swings, etc.)		
3. Economic insecurity/poverty		
4. Unemployment		
5. Housing problems (e.g. unable to pay rent, poor living conditions, don't have own home)		
6. Land issues (e.g. lack of access to land, unable to return to own land, etc.)		
7. Living as an internally displaced person		
8. Difficulty in meeting basic everyday needs (e.g. water, food, electricity, sanitation, clothing)		
9. Lack of access to healthcare		
10. Lack of access to education (for self or children)		
11. Problems with partner		
12. Other family and relationship problems		
13. Abuse/bullying from community members		
14. Loneliness		
15. Addictions (e.g. alcoholism)		
16. Domestic violence		
17. Threats (e.g. death threats, threats against family members)		
18. Other (please specify)		

5. Life Today

**Do you feel safe in your community?**				
1. Never	2. Occasionally	3. Sometimes	4. Most of the time	5. Always

**Do you feel able to ask for help when you need it?**				
1. Never	2. Occasionally	3. Sometimes	4. Most of the time	5. Always

**In general, how would you rate your health?**				
1. Poor	2. Fair	3. Good	4. Very good	5. Excellent

How would you rate your quality of life?				
1. Poor	2. Fair	3. Good	4. Very good	5. Excellent

References

Berntsen, D., & Rubin, D.C. (2006). The centrality of event scale: A measure of integrating a trauma into one's identity and its relation to post-traumatic stress disorder symptoms. *Behaviour Research & Therapy*, *44*(2), 219–231. https://doi.org/10.1016/j.brat.2005.01.009

Resilience Research Centre (2016). *The Resilience Research Centre Adult Resilience Measure (RRC-ARM): User's manual – Research, May 2016*. https://cyrm.resilienceresearch.org/files/ArchivedMaterials.zip
